# Signal transduction in light–oxygen–voltage receptors lacking the adduct-forming cysteine residue

**DOI:** 10.1038/ncomms10079

**Published:** 2015-12-09

**Authors:** Estella F. Yee, Ralph P. Diensthuber, Anand T. Vaidya, Peter P. Borbat, Christopher Engelhard, Jack H. Freed, Robert Bittl, Andreas Möglich, Brian R. Crane

**Affiliations:** 1 Department of Chemistry and Chemical Biology, Baker Laboratory, Cornell University, Ithaca, New York 14853, USA; 2 Biophysikalische Chemie, Institut für Biologie, Humboldt-Universität zu Berlin, Berlin 10115, Germany; 3 National Biomedical Center for Advanced ESR Technology, Cornell University, Ithaca, New York 14853, USA; 4 Fachbereich Physik, Institut für Experimentalphysik, Freie Universität Berlin, Berlin 14195, Germany; 5 Lehrstuhl für Biochemie, Universität Bayreuth, Bayreuth 95400, Germany

## Abstract

Light–oxygen–voltage (LOV) receptors sense blue light through the photochemical generation of a covalent adduct between a flavin-nucleotide chromophore and a strictly conserved cysteine residue. Here we show that, after cysteine removal, the circadian-clock LOV-protein Vivid still undergoes light-induced dimerization and signalling because of flavin photoreduction to the neutral semiquinone (NSQ). Similarly, photoreduction of the engineered LOV histidine kinase YF1 to the NSQ modulates activity and downstream effects on gene expression. Signal transduction in both proteins hence hinges on flavin protonation, which is common to both the cysteinyl adduct and the NSQ. This general mechanism is also conserved by natural cysteine-less, LOV-like regulators that respond to chemical or photoreduction of their flavin cofactors. As LOV proteins can react to light even when devoid of the adduct-forming cysteine, modern LOV photoreceptors may have arisen from ancestral redox-active flavoproteins. The ability to tune LOV reactivity through photoreduction may have important implications for LOV mechanism and optogenetic applications.

## Abstract (web summary)

Light-oxygen-voltage receptors sense blue light through the photochemical generation of a covalent adduct between a flavin-nucleotide chromophore and a strictly conserved cysteine residue. Here, the authors show that these proteins can react to light even when devoid of the adduct-forming cysteine.

## Graphical abstract

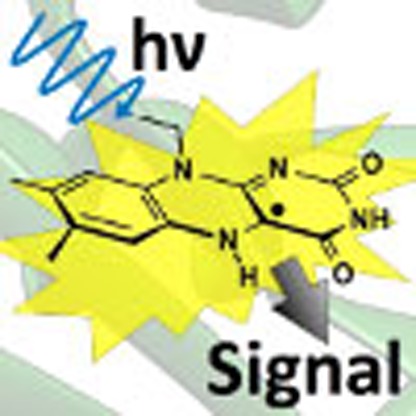

Flavin-binding proteins widely occur across all kingdoms of life where they play vital roles in many different aspects of metabolism^1^,^2^,^3^. Beyond their crucial function in redox catalysis, flavin-binding proteins also serve as signal receptors for redox potential, partial oxygen pressure and blue light^1^,^2^,^3^. Flavin-based sensory photoreceptors fall into three major classes: light, oxygen and voltage sensing (LOV) proteins; BLUF proteins (sensors of blue light using flavin adenine dinucleotide (FAD)) and photolyases/cryptochromes (PL/CRY)^1^,^2^,^3^. The utility of flavin as a chromophore derives from the ability of light to facilitate interconversion between excited states and three ground oxidation states: namely the fully oxidized quinone, the partially reduced semiquinone radical and the fully reduced hydroquinone (HQ). Of these, the oxidized quinone-bound form often serves as the dark-adapted state because of its strong absorption in the blue spectral region.

LOV photoreceptors occur throughout archaea, bacteria, protists, fungi and plants, and regulate phototropism, chloroplast movement, stomatal opening, virulence, stress response, circadian rhythms and other physiological responses^1^,^2^,^3^,^4^. In the well-characterized LOV photocycle^1^,^2^,^3^, blue-light absorption drives formation of a covalent adduct between a strictly conserved, active-site Cys residue and the C4a atom of the flavin isoalloxazine ring (either FAD; or flavin mononucleotide (FMN))^2^,^5^ (Fig. 1a). In at least one case, a neutral flavin semiquinone radical intermediate has been detected^6^, suggesting that bond formation proceeds via rapid reduction of the flavin by the Cys thiol to form a neutral radical pair (FMNH·—·S-Cys) and subsequent radical recombination^5^,^6^,^7^; however, more recent spectroscopic studies could not identify build-up of such a species on the tens of ns-to-μs time scale and thereby concluded that if a flavin radical intermediate forms it must react faster than the rate by which it is produced^8^. Nonetheless, effective reduction of the flavin ring, by either adduct or radical formation greatly increases the pK_a_ of N5, thus promoting its protonation^9^. In response to this protonation, a nearby glutamine residue, conserved in most LOV proteins, undergoes a 180° flip of its amide side chain to adjust hydrogen-bonding interactions^10^,^11^,^12^. Additional changes in hydrogen bonding propagate through the core α/β PAS-domain fold of the LOV domain to N-cap and C-cap regions that pack against the β-sheet on the side opposing the flavin-binding pocket^1^,^2^,^3^. Alteration of the structure and dynamics of these cap regions affect oligomeric state and the activity of output modules^1^,^3^. However, it is not clear whether adduct formation itself is the dominant factor in generating downstream signalling responses, or if N5 protonation alone can suffice. Despite the paramount importance of the conserved cysteine residue in the canonical LOV photocycle, at least some LOV photoreceptors apparently retain biological activity even after substitution of the Cys thiol for non-reactive side chains^12^,^13^,^14^,^15^. Although a rationalization of these findings has remained elusive, it is well-established that LOV domains devoid of this cysteine can undergo photoreduction to a neutral semiquinone (NSQ) state with N5 protonated^16^,^17^,^18^,^19^,^20^, thus raising the question as to whether the NSQ triggers downstream signalling.

Taking the two well-characterized LOV photoreceptors Vivid (VVD)^12^,^13^ and YF1^21^, we demonstrate that the NSQ state indeed mediates wild-type (WT)-like signalling responses and that LOV photoreceptors devoid of the conserved adduct-forming cysteine are thus fully capable of light-dependent signal transduction. Furthermore, sequence analyses reveal natural LOV-like proteins that lack the adduct-forming cysteine residue. We demonstrate that one such protein from archaeal halobacteria binds flavin nucleotides and undergoes chemical- and photo-reduction processes that couple to changes in protein conformation. Our results lend new insight into photoreception and signal transduction by LOV photoreceptors, bear on LOV domain application as optogenetic tools^2^,^22^ and suggest an intriguing possibility for the evolutionary origin of the widespread LOV photoreceptor family.

## Results

### Photoconversion of VVD lacking the adduct-forming Cys

The replacement of the active-site Cys108 of VVD by Ala was produced in the context of VVD-II, which lacks the first 36 N-terminal unstructured residues and carries two other substitutions (M135I:M165I) that are known to stabilize the VVD light-adapted state^12^,^23^. The Met substitutions extend the adduct lifetime of VVD-II by 10-fold compared with WT VVD and upshift the flavin redox potential by removing two electron-rich sulfur residues that pack against the re-face of the flavin^12^,^23^. Accordingly, VVD-II purifies from overexpression in *Escherichia coli* as a pale green protein indicative of partial reduction to the NSQ (Supplementary Fig. 1b). Introduction of the C108A substitution into VVD-II yielded VVD-III (VVD Δ36, C108A:M135I:M165I), which purifies as a much darker green protein than VVD-II (Supplementary Fig. 1b). Different flavin redox states separate when the protein is passed through a size-exclusion chromatography (SEC) column (Supplementary Fig. 1d), with the blue-coloured, NSQ-containing protein eluting as a dimer and the yellow-coloured, oxidized protein eluting as a monomer. Intriguingly, this behaviour is highly reminiscent of the homodimerization of WT VVD on blue-light-induced adduct formation^12^,^24^. On SEC, the adduct form of WT VVD elutes at a larger volume due to a rapidly exchanging equilibrium between dimer and monomer^24^. Light-induced VVD dimerization is critical for the protein to mediate photoadaptation in *Neurospora*^11^,^12^.

**Figure 1:**
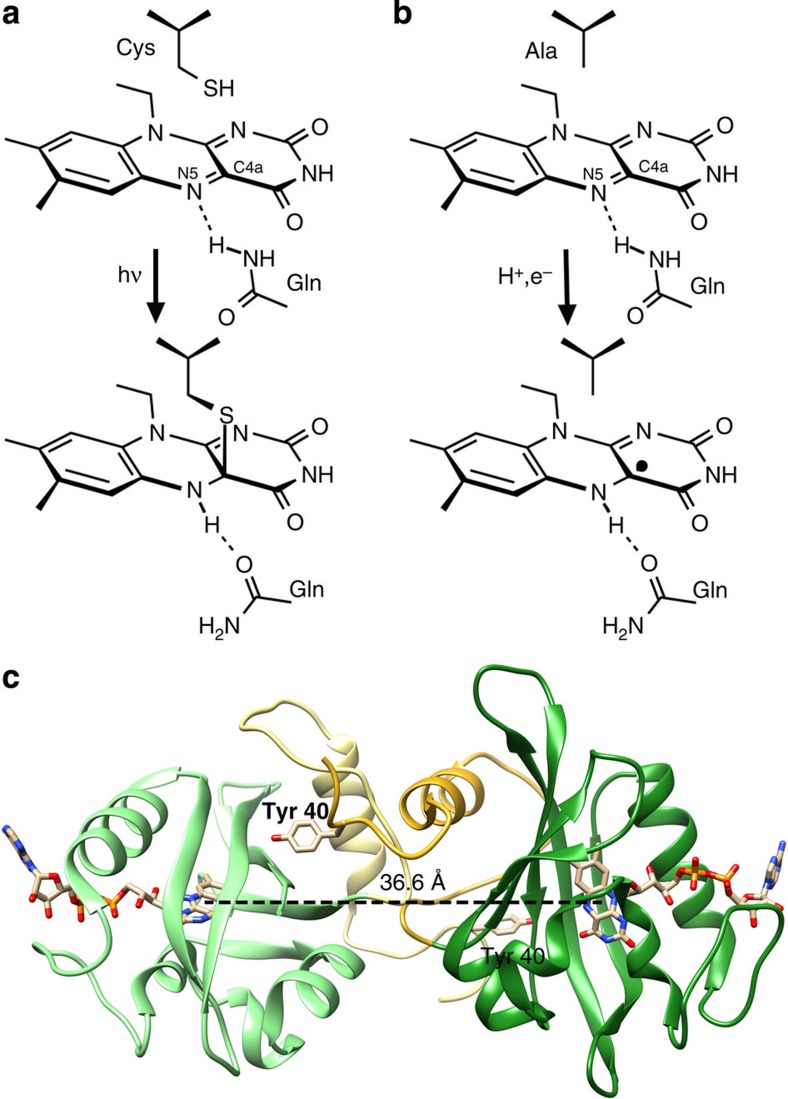
Photochemistry of LOV photoreceptors and structure of VVD. (**a**) In canonical LOV photoreceptors, light excitation of the flavin leads to a covalent adduct between the C4a atom of the flavin ring and an active-site Cys thiol (residues 108 and 62 in VVD and YF1, respectively). Coincident protonation of the flavin atom N5 induces a flip of the amide side chain of a conserved Gln residue (residues 182 and 123 in VVD and YF1, respectively). Resultant changes in hydrogen bonding propagate through the LOV photoreceptor, for example, to an N-cap region in the case of VVD. (**b**) The absence of the adduct-forming cysteine promotes photoreduction of the LOV flavin to the neutral semiquinone (NSQ). As N5 of the NSQ is also protonated, signals could be relayed in a manner corresponding to that in the cysteinyl adduct in **a**. (**c**) Structure of the light-adapted VVD dimer (3RH8)^12^. The flavin rings (tan) in the constituent subunits (dark and light green ribbons) are separated by ∼37 Å at their centroids. An exchange of N-terminal latches associates the N-caps (yellow), with Tyr40 making a key contact across the dimer interface.

**Figure 2:**
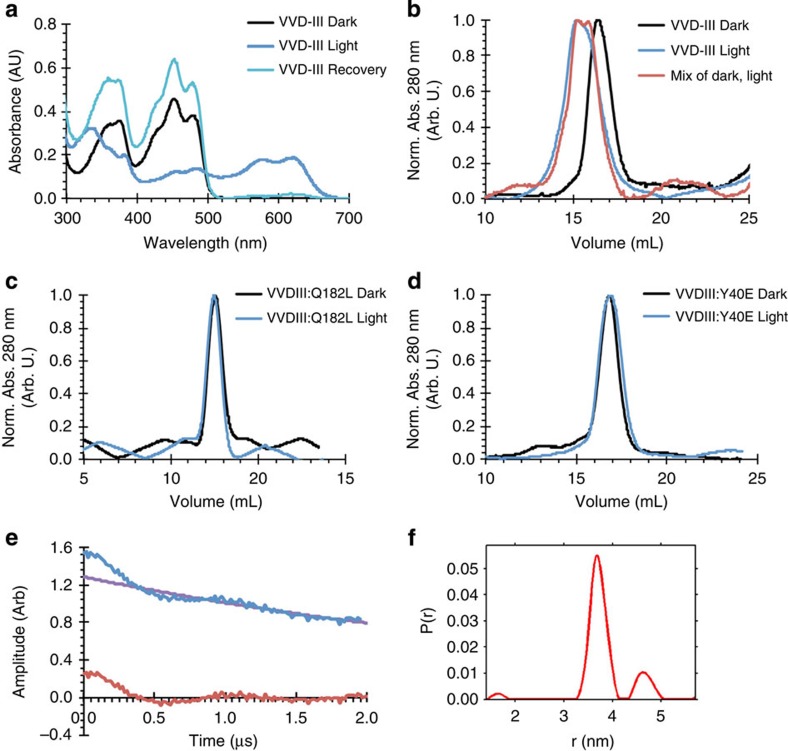
Photoreduced VVD-III forms the same light-adapted dimer as wild-type VVD. (**a**) Absorption spectra of VVD-III in its dark-adapted state (black) and neutral semiquinone state after photoreduction for 5 min with a 448-nm 30 mW laser (dark blue) followed by reoxidation after 170 min at ambient conditions (light blue). (**b**) Size-exclusion chromatography (SEC) of dark-adapted (black) and light-adapted VVD-III (blue) indicates dimer formation in the NSQ state. A 1:1 mixture of dark-adapted and light-adapted states produces intermediate peaks (red), indicative of the exchangeable nature of the VVD dimer and perhaps radical equilibration on the SEC time scale. (**c**,**d**) SEC traces of dark-adapted (black) and light-adapted (blue) VVD-III:Q182L (**c**) and VVD-III:Y40E (**d**) indicate no change in the oligomeric state of either protein on photoreduction to the NSQ. Q182L and Y40E traces have different baselines because different SEC columns were used. (**e**,**f**) Magnetic dipolar coupling between two radical flavin states was detected by DEER spectroscopy in illuminated VVD-III. (**e**) Raw time-domain DEER data (blue) with baseline (purple) and after baseline-subtraction (red) reveal a clear dipolar oscillation that produces a sharply peaked distance distribution *P*(*r*) of the separated spins at ∼37 Å (**f**). The smaller peak in *P*(*r*) at longer distance is a reconstruction artifact. The spin separation agrees well with that predicted by the light-adapted dimer of VVD (Fig. 1c).

Exposure to a 448-nm laser (30 mW) reduces the VVD-III FAD quinone to the NSQ over the course of 5 min (Fig. 2a and Supplementary Fig. 2a). The FAD then recovers completely to the oxidized form within ∼3 h in the dark under aerobic conditions (Fig. 2a and Supplementary Fig. 2b). By following the loss of oxidized flavin fluorescence and formation of the NSQ by absorption spectroscopy, the relative quantum efficiency of reduction compared with adduct formation in WT VVD was determined to be 0.11±0.07. The unprotonated anionic semiquinone was not observed on this time scale, and two isosbestic points (347 and 497 nm) in the forward photoreduction indicate the presence of only oxidized FAD and the NSQ (Supplementary Fig. 2a). Formation of the NSQ implies that flavin N5 protonates readily on reduction of the isoalloxazine ring (Fig. 1b). The mechanism for photoreductive quenching of VVD-III to the NSQ is currently unclear, but does not appear to require oxidation of aromatic residues within the protein as substitution of all aromatic residues to Phe has little effect on the rate of VVD-III photoreduction.

When subjected to SEC, the light-adapted NSQ state of VVD-III elutes as a dimer, whereas the dark-adapted oxidized quinone state remains monomeric (Fig. 2b). Mixtures of the dark and light states elute at an intermediate volume, which suggests that either a NSQ subunit can associate weakly with a quinone subunit or that these oxidation states rapidly equilibrate within the population (Fig. 2b). Substituting the conserved Gln182 that interacts with flavin N5 to Leu (*cf.* Fig. 1a,b) does not affect photoreduction but completely prevents dimerization (Fig. 2c).

To confirm that the NSQ-containing VVD-III dimer has the same structure as that of the Cys-adduct dimer, we introduced the Y40E mutation (VVD-III:Y40E), which disrupts the dimeric interface necessary for signalling^12^. Indeed, introduction of Y40E in VVD-III:Y40E abrogates light-induced dimer formation, and both the light-adapted NSQ and dark-adapted oxidized quinone states elute from SEC as monomers (Fig. 2d). To further investigate the structure of the VVD-III light-adapted dimer, we applied double electron–electron resonance (DEER) spectroscopy, which measures the magnetic dipolar coupling between remote electron spins and hence their distance of separation. Modulation of the spin-echo amplitude in photoreduced VVD-III shows a substantial oscillation characteristic of two interacting spins (Fig. 2e). The derived distance distribution features a sharp peak at ∼37 Å (Fig. 2f), which matches the expected separation of the flavin radicals based on the crystal structure of the light-adapted VVD dimer^12^ (*cf.* Fig. 1c). Thus, photoreduction of VVD-III to the NSQ produces the same light-adapted dimer as the cysteinyl-flavin adduct in the WT protein.

### Signal transduction in YF1 lacking the adduct-forming Cys

To assess whether the NSQ state can generally activate signalling in LOV proteins, we investigated photoreduction of the LOV histidine kinase YF1, which derives from the fusion of the LOV module of *Bacillus subtilis* YtvA to the histidine-kinase effector module of *Bradyrhizobium japonicum* FixL^21^. As a well-characterized paradigm, YF1 is emblematic of numerous natural proteins in which a LOV or PAS module regulates a histidine kinase^25^,^26^. Compared with VVD, YF1 is of different origin (prokaryotic versus eukaryotic), utilizes FMN instead of FAD, and possesses different effector output^27^,^28^. Like VVD, the C62A variant of YF1 undergoes photoreduction with blue light to the NSQ without population of the anionic semiquinone radical (Supplementary Fig. 3a). Addition of reductants, such as TCEP (Tris(2-carboxyethyl)phosphine hydrochloride), greatly enhances the rate and yield of NSQ formation. Once blue-light illumination ceases, the NSQ oxidizes back to the quinone state in a largely monophasic process over the course of several hours (Supplementary Fig. 3b).

**Figure 3:**
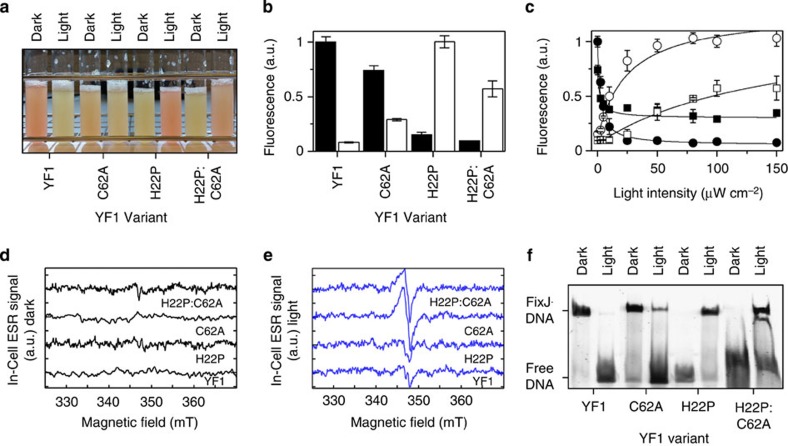
Activity measurements of YF1 variants. (**a**) Light-regulated activity of YF1 variants was assessed *in vivo* in the pDusk-DsRed reporter system^28^. Cultures of YF1 WT and variants C62A, H22P and H22P:C62A were grown in 5 ml under dark and blue-light conditions (470 nm, 100 μW cm^−2^). DsRed expression is evident by red colouration of cultures. (**b**) DsRed fluorescence per OD_600_ was measured for the cultures in **a** under blue-light (white bars) and dark conditions (black bars). (**c**) The experiment in **b** was repeated at varying blue-light intensities (470 nm) between 0 and 150 μW cm^−2^. YF1 (filled circle) and YF1 C62A (filled square) have nearly the same light-dose dependencies with half-maximal light doses, ED_50_, of (2.1±0.6) μW cm^−2^ and (2.0±0.7) μW cm^−2^, respectively. By contrast, YF1 H22P (empty circle) shows a higher half-maximal dose of (24.6±5.8) μW cm^−2^ that is increased to above 100 μW cm^−2^ for YF1 H22P:C62A (empty square). The precise ED_50_ value for YF1 H22P:C62A cannot be determined due to cytotoxicity of blue-light doses higher than 150 μW cm^−2^. All data in **b**,**c** represent mean±s.d. of biological triplicates. (**d**,**e**) *E. coli* cultures from **a** were analysed by whole-cell ESR spectroscopy under the same conditions used in **a**–**c**. Spectra recorded for dark-adapted (**d**) or blue-light-adapted (**e**) cultures were corrected for the *E. coli* background ESR signal. Significant population of flavin radicals above background is only observed for the cysteine-devoid variants YF1 C62A and YF1 H22P:C62A under blue-light illumination. (**f**) *In vitro* activity measurements of YF1 variants by electrophoretic mobility shift assays (EMSA). In its dark-adapted state, YF1 phosphorylates FixJ, which then binds a rhodamine-labelled DNA substrate, retarding migration of the resultant phospho-FixJ:DNA complex in the polyacrylamide gel. In its light-adapted state, YF1 acts as a phosphatase, FixJ is not phosphorylated and no DNA upshift is observed. The inverter variant YF1 H22P shows the opposite behaviour with an upshift under blue light but not in the dark.

To assess whether photoreduction of YF1 C62A to the NSQ suffices to elicit WT-like downstream signalling responses, we capitalized on efficient assays that allow probing YF1 signalling *in vivo* and *in vitro*. Combined with the cognate response regulator FixJ, YF1 forms a two-component system that drives blue-light-repressed gene expression of the red-fluorescent reporter DsRed^21^,^28^. When incubated in the dark, *E. coli* cultures harbouring the pDusk plasmid^28^ display readily discernible DsRed fluorescence due to FixJ phosphorylation by YF1; when cultures are incubated under saturating blue light (100 μW cm^−2^, 470 nm), fluorescence is diminished by about 15-fold owing to dephosphorylation of FixJ by YF1 (Fig. 3a,b). Replacement of Cys62 in YF1 by alanine slightly reduces DsRed expression in the dark; but, similar to WT, blue-light illumination induces a large (60%) repression of the DsRed fluorescence signal (Fig. 3a,b). YF1 and YF1 C62A show remarkably similar light-dose dependencies with half-maximal light doses, ED_50_, of (2.1±0.6) μW cm^−2^ and (2.0±0.7) μW cm^−2^, respectively (Fig. 3c). Evidently, the C62A variant of YF1 is still capable of mediating light-dependent signal transduction *in vivo,* albeit the response is partially attenuated.

Removal of the adduct-forming cysteine in LOV proteins not only promotes photoreduction and enhances the flavin fluorescence^29^, but also renders the flavin a photosensitizer for the generation of singlet oxygen^30^,^31^,^32^. Blue-light-driven generation of reactive oxygen species (ROS) might thus interfere with the two-component system by causing rupture of the labile acid anhydride bond in phospho-FixJ and concomitant deactivation of gene expression. To confirm that YF1 C62A does not incapacitate FixJ through ROS production, we investigated the H22P mutant of YF1 that shows an inverted response to light^27^,^33^. In contrast to the WT, blue-light illumination of the H22P variant stimulates reporter gene expression by about 10-fold, that is, blue light induces a gain-of-function (Fig. 3a–c). Introduction of the C62A mutation into the YF1 H22P inverter variant has no effect on the DsRed expression levels in the dark. When incubated under blue light, DsRed expression levels for YF1 H22P:C62A increase about sixfold to ∼60% of the value seen for YF1 H22P in the light. Interestingly, YF1 H22P:C62A showed a lower light sensitivity than YF1 H22P, and higher light doses were required for saturation (Fig. 3a–c). Thus, YF1 H22P:C62A is also capable of blue-light signal transduction without the active-site Cys residue, and because this variant increases gene expression, this effect cannot be due to ROS generation.

We sought evidence that regulation of gene expression by the cysteine-devoid YF1 variants correlates with population of the NSQ *in vivo*, and conducted whole-cell electron-spin resonance (ESR) spectroscopy on the above *E. coli* cultures. Dark-grown and light-grown cultures of YF1 and YF1 H22P, either with or without the adduct-forming cysteine residue, were rapidly frozen in liquid nitrogen. Continuous-wave ESR spectra were recorded and corrected for signals arising from endogenous *E. coli* cell constituents as determined from control cultures not expressing any YF1 variants (Fig. 3d,e). The ESR spectra of YF1 and YF1 H22P with the adduct-forming Cys residues intact revealed no significant accumulation of flavin radicals above background under either dark or light conditions. By contrast, the cysteine-devoid variants YF1 C62A and YF1 H22P:C62A produced significantly elevated levels of flavin radical species under blue light but not in the dark. Note that the width of these signals (∼10 G) is consistent with the broadening expected from the hyperfine interactions of flavin radicals (Fig. 3e). These data suggest that photoreduction to the NSQ states of YF1 C62A and YF1 H22P:C62A readily takes place inside living *E. coli* cells and correlates with downstream signalling.

To further verify these *in vivo* effects, we also directly measured the ability of YF1 to regulate FixJ binding to DNA. Once YF1 phosphorylates FixJ, phospho-FixJ assembles into a homodimer and binds to the DNA substrate. In electrophoretic mobility shift assays (EMSA), the resultant complex migrates more slowly than the free DNA substrate (Fig. 3f). In the dark, YF1 has net kinase activity, FixJ is phosphorylated and the DNA is in complex; by contrast, under blue light, YF1 has net phosphatase activity^21^, FixJ is dephosphorylated, and no DNA gel shift is observed. For YF1 C62A, an upshift of the DNA band is seen in the dark, indicating activity as a net kinase. Under blue-light conditions, most of the DNA is present in its free form and only a small portion is in complex with FixJ. As found *in vivo*, YF1 H22P shows the inverted signal response compared with YF1—an upshift of the DNA band indicative of FixJ phosphorylation under blue-light illumination, but not in the dark. Interestingly, DNA binding of FixJ induced by H22P:C62A was only observed in the light and in the presence of a ROS scavenger system (consisting of catalase, glucose and glucose oxidase). Apparently, under these conditions, YF1 H22P:C62A does generate ROS that can interfere with the FixJ response. Taken together, the *in vitro* results are in agreement with the *in vivo* findings and indicate that cysteine-devoid YF1 variants are capable of signal transduction on photoreduction to the NSQ.

### Natural LOV-like proteins lacking the adduct-forming Cys

**Figure 4:**
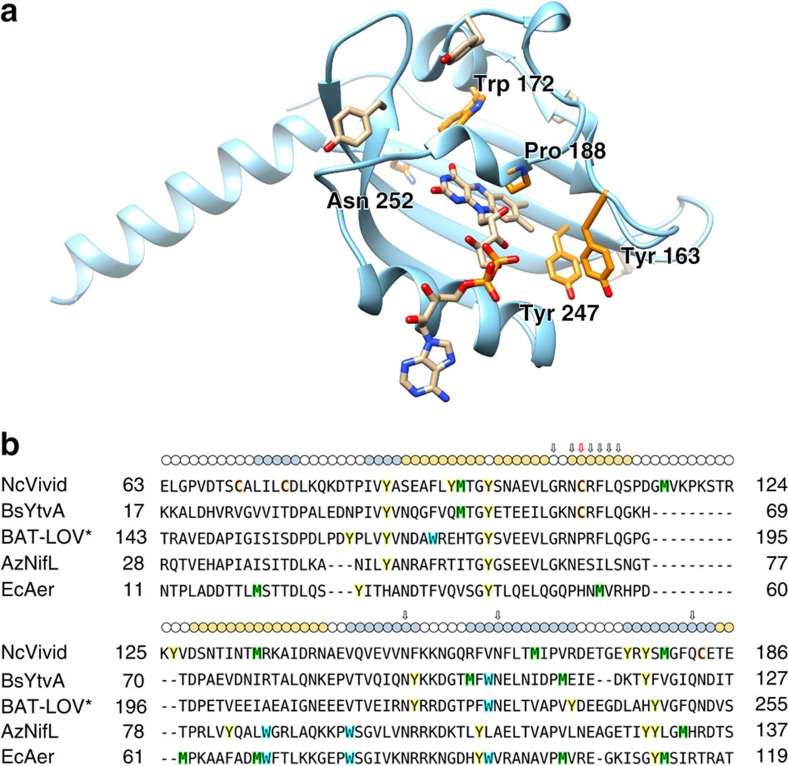
Structural model and sequence comparison for BAT-LOV*. (**a**) Homology model of BAT-LOV* bound to FAD based on sequence similarity to YtvA. Tyr163 and Tyr247 were changed to Phe in BAT-II, with Trp172 also changed to Phe in BAT-III. The P188C substitution (but not N252C) affects photoreduction yields. Three Tyr residues remote from the flavin are also shown. (**b**) Sequence alignment of VVD, BAT-LOV*, YtvA, *A. vinelandii* NifL and *E. coli* Aer. Tyr residues (yellow), Trp (blue), Cys (orange) and Met (green) are highlighted; circles above the alignment indicate the secondary structure in VVD with β sheets in blue and α helices in orange; arrows denote LOV-conserved residues, with the adduct-forming Cys in VVD and YtvA marked by a red arrow.

**Figure 5:**
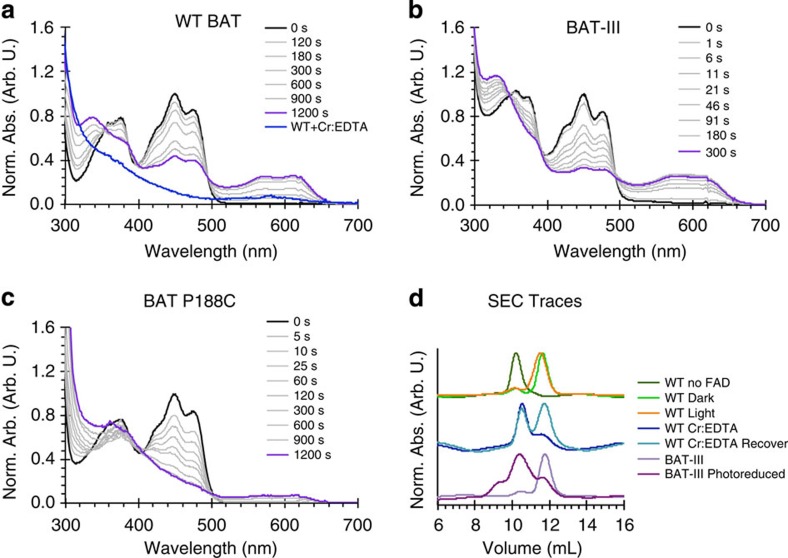
Photochemistry of BAT-LOV*. Photoreduction of recombinantly expressed, FAD-reconstituted (**a**) BAT-LOV* and (**b**) BAT-III (Y163F:W172F:Y247F). BAT-LOV* reduces slowly to the NSQ, whereas BAT-III reduces to the NSQ much more rapidly. Chemical reduction of BAT-LOV* with Cr:EDTA (blue) forms the HQ directly (**a**). (**c**) BAT P188C photoreduces to the HQ with little NSQ intermediate. Experiments in **a**–**c** were carried out on similar protein concentrations. See Supplementary Fig. 6a for photoreduction rate constants. (**d**) SEC elution profile of BAT-LOV* and variants. Ambient light exposure of BAT-LOV* produces no shift on SEC (WT Dark compared with WT Light); however, chemical reduction (WT Cr:EDTA) results in a shift to an extended conformation that is similar to that of the protein stripped of flavin by anion exchange chromatography (WT no FAD). Reoxidation partially reforms the compact state (WT Cr:EDTA Recover). Photoreduction of BAT-III forms a state similar to that observed for reduced WT BAT-LOV* (BAT-III Photoreduced). Multi-angle light scattering confirmed that BAT-LOV* remained a monomer in the dark and with ambient light exposure; loss of FAD binding also does not alter the oligomeric state of BAT-III (Supplementary Fig. 8a,b).

The observation that photoreduction elicits WT-like signalling responses in two different LOV proteins devoid of the adduct-forming Cys residue suggested that related proteins might occur naturally. A BLAST sequence search in Genbank for LOV-like proteins that contain all residues strictly conserved among LOV domains (see Methods) except for the adduct-forming cysteine revealed ca. 70 entries with this Cys replaced by one of several other residues, including Ala, His and Pro (Fig. 4b and Supplementary Fig. 4a). Cysteine-devoid LOV-like domains, denoted LOV*, are found in proteins with varied architectures (Supplementary Data 1). Among these proteins, we focused on a LOV* domain from a halobacterial archaea that has relatively close homology to VVD and contains an unreactive Pro residue in place of the adduct-forming Cys. This LOV* domain is a component of a much larger protein known as bacterio-opsin activator (BAT)^34^, which also contains a response-regulator receiver domain, a GAF domain and a helix-turn-helix DNA-binding module. Notably, BAT-LOV* contains no Cys or Met residues, with the position of the adduct-forming Cys occupied by Pro (Fig. 4b). When overexpressed in *E. coli*, BAT-LOV* bound modest amounts of flavin but could be reconstituted with either FAD, FMN or riboflavin after incubation with excess cofactor (Supplementary Fig. 5b). Although BAT-LOV* does not contain an insertion usually found to accommodate the adenosine moiety of FAD-binding LOV domains^11^ (Fig. 4b), reconstitution with FAD yielded no noticeable difference in photoreduction or behaviour on SEC compared with reconstitution with FMN (Supplementary Figs 5c and 6a). On photoreduction, reconstituted BAT-LOV* accumulated the NSQ with low yield compared with VVD-III (Fig. 5a and Supplementary Fig. 6a). Moreover, photoreduction is nearly two orders of magnitude slower than for VVD-III, and the corresponding rates can only be moderately increased by adding external reductive quenchers, such as dithiothreitol (DTT) (Fig. 5a and Supplementary Fig. 6a). A small lag phase for BAT-LOV* photoreduction, not seen with VVD-III, depends on the presence of oxygen (Supplementary Fig. 6b). Low fluorescence quantum yields for BAT-LOV* compared with VVD-III (Table 1) indicate a much reduced lifetime of the initial S_1_ photo-excited state, possibly owing to rapid reversible reductive quenching from internal redox-active residues. Inspection of a BAT-LOV* homology model (Fig. 4a) identified two Tyr residues and one Trp residue that reside closer to the flavin than any aromatic residues in VVD (Fig. 4b). Substitution of the Tyr residues to Phe (BAT-II) produced little change in fluorescent yields or NSQ accumulation (Table 1). However, additional substitution of Trp172 to Phe (BAT-III) caused a large increase in fluorescent lifetime and photoreduction rates that are similar to those of VVD-III (Table 1, Fig. 5b, Supplementary Fig. 6a). Notably, most BAT-LOV* homologues contain a Phe at the position of Trp172 (Supplementary Fig. 4a), and thus would be expected to have similar photoreduction yields as the BAT-III variant examined here.

Although photoreduction of WT BAT-LOV* is ineffective, the protein readily undergoes complete chemical reduction to the HQ (Fig. 5a and Supplementary Fig. 7). Reduced BAT-LOV* elutes on SEC with a profile shifted from that of the oxidized dark-state protein (Fig. 5d). When flavin is removed from BAT-LOV*, a similar shift on SEC results, suggesting that reduction destabilizes flavin binding. Nevertheless, the reduced flavin remains loosely associated with BAT-LOV* as the compact state and spectrum for the quinone-bound protein partially recover after reoxidation in air (Fig. 5d and Supplementary Fig. 7). WT BAT-LOV* does not shift on SEC with light exposure owing to inefficient photoreduction; however, the more readily photoreduced BAT-III elutes at the same position as chemically reduced BAT-LOV*, while retaining a monomeric molecular mass (Fig. 5d and Supplementary Fig. 8a,b). Thus, both reduction processes influence protein conformation and destabilize flavin binding in the isolated LOV* domains. BAT-III recovers fully after reoxidation in aerobic solution, producing a spectrum for oxidized bound flavin (Supplementary Fig. 6d).

We attempted to convert BAT-LOV* to a traditional LOV mechanism by mutating the active-site Pro residue to Cys. The P188C variant does not undergo conversion to a traditional adduct state with its characteristic 390-nm absorption peak, but the variant is much more readily photoreduced than WT, rivalling the reactivity of BAT-III (Fig. 5c and Supplementary Fig. 6a). Interestingly, the P188C appears to form the HQ directly with little NSQ intermediate observed on this time scale. Unlike the C4a adduct in canonical LOV domains, the light-adapted state of the P188C variant can be rapidly chemically oxidized to the quinone state, after which the flavin partially dissociates from the protein (Supplementary Fig. 9). A control Cys substitution distant from the flavin (N252C) increases photoreduction only marginally (Supplementary Fig. 6a).

## Discussion

Cysteine-adduct formation causes substantial changes to the LOV flavin pocket that include electronic redistribution in the cofactor, bond strain and protonation of the flavin N5 atom^2^,^3^,^35^. It has been challenging to assign the relative impact of these various factors on signal propagation. In this study, we partially separate these events and directly evaluate the role of the cysteinyl-flavin bond in signalling. As we show for the paradigm LOV photoreceptors VVD and YF1, blue light promotes reduction to the NSQ state in the absence of the adduct-forming cysteine. Corresponding photoreduction has been reported for several other LOV domains in which the adduct-forming cysteine has been replaced^16^,^17^,^18^,^20^,^36^. We now demonstrate that LOV NSQ states are biologically functional in that they elicit downstream signalling responses largely equivalent to those for the Cys-adduct states of the parental photoreceptors. In particular, photoreduced VVD-III associates into the same light-adapted dimer as WT VVD. Structural studies of VVD in its dark-adapted and signalling states indeed suggest that conformational changes important for promoting dimerization depend on N5 protonation^12^. These results are further borne out in YF1 C62A variants that on photoreduction show qualitatively the same light responses as the corresponding Cys-containing receptors, both in the original context and in the context of the H22P variant that inverts the light response. We thus conclude that flavin reduction and protonation of the flavin N5 are sufficient for signal transduction in these LOV domains. Our rather unexpected findings account for several puzzling observations in LOV photoreceptors. For example, the residual light responsiveness of the C108A variant of VVD in repressing downstream gene expression^13^ can now be explained by photoreduction to the NSQ state. A similar mechanism is likely at play in variants of *Chlamydomonas reinhardtii* phototropin 1, which were found to elicit light responses even though the adduct-forming Cys residues of the two LOV domains had been replaced by mutagenesis (C57S:C250A)^14^,^15^.

The present results also bear on mechanistic studies of LOV photoreceptors and their biotechnological application. Often, cysteine knock-out variants are used as negative controls in LOV photoreceptor studies; given the likelihood of activation by photoreduction, responses from these variants should be carefully considered. Similarly, LOV domains devoid of their adduct-forming cysteine have found frequent use as fluorescent protein tags and as photosensitizers for the generation of ROS^29^,^30^. As the present results reveal, these model LOV domains may still populate a signalling state through NSQ formation that could have functional consequences, even in heterologous hosts. By contrast, in other scenarios signal transduction by Cys-less LOV variants could be desirable: for example, removal of the adduct-forming cysteine will lower the absolute light sensitivity of LOV photoreceptors as shown for YF1 H22P:C62A (Fig. 3c), which may be of use in optogenetic applications^22^,^37^. As demonstrated by BAT-III, photoreduction yields may be tuned by the location of residues capable of reductively quenching the flavin excited-state. Removal of the adduct-forming cysteine hence provides an avenue towards modulating the light-driven forward reaction, which is challenging to perturb in canonical LOV domains^38^. Nonetheless, we reiterate that cysteine-devoid LOV variants come with enhanced fluorescence and ROS generation^29^,^30^.

Our results reveal commonalities among flavin-based photo- and redox receptors that could reflect evolutionary relationships among them. In the canonical LOV photocycle, a flavin excited triplet state reacts with the thiol group of a conserved cysteine residue^2^,^3^,^35^. Bond formation likely proceeds via a redox process, as supported by detection of a transient flavin NSQ in *C. reinhardtii* phot1 LOV1 (ref. 6), by indirect arguments from magnetic resonance experiments^39^,^40^, and by the general efficacy of flavin photoreduction in the cysteine-devoid variants^16^,^17^,^18^,^20^,^36^. Thus, the NSQ is a likely intermediate in generating the adduct. The BAT-LOV* P188C variant demonstrates that Cys at the adduct-forming position is an effective electron donor to the photo-excited flavin. LOV signalling through the NSQ state has intriguing parallels to signal transduction in the other flavin-based photoreceptor classes cryptochrome and BLUF^1^,^2^,^3^. Although controversial, there is strong evidence that the signalling state of cryptochromes involves reduction of the FAD to either the NSQ or anionic semiquinone states^1^,^3^,^41^. While the details of BLUF photochemistry are still under intense debate, a NSQ state may be populated transiently during the photocycle as part of a radical-pair intermediate between the flavin and a conserved Tyr (FADH·—·O-Tyr)^42^. Interestingly, removal of the conserved Tyr allows efficient photoreduction of the BLUF protein to the NSQ state^43^; moreover, this NSQ state regulates the activity of an adenylate-cyclase effector, albeit with inverted polarity and reduced dynamic range compared with WT.

Changes in flavin redox state affect conformation and flavin binding in the naturally Cys-less BAT-LOV*. BAT regulates expression of bacteriorhodopsin, a light-driven proton pump expressed in halobacteria under conditions of high light intensity and low oxygen levels^34^. A direct response to light by BAT has been suggested^34^ but has not been definitively established. BAT-LOV* shares some relationship to the flavin-binding oxygen sensor proteins *E. coli* Aer^44^ and *Azotobacter vinelandii* NifL, but its sequence is more similar to LOV domains, such as those of VVD or YF1 (Fig. 4b). Aer, which senses O_2_ indirectly by a redox response to the membrane potential^44^, can also act as a photoreceptor^45^. BAT retains the conserved Gln for responding to protonation changes at N5 and indeed changes conformation on flavin reduction. The isolated BAT-LOV* domain studied here cannot be effectively photoreduced because its excited-state lifetime is too short, owing to reductive quenching by neighbouring aromatic residues. However, substitution of an unconserved Trp to its more typical occurrence of Phe generates photoreduction yields that rival those of VVD-III. Thus, for both BAT and possibly Aer, either chemical or light-driven flavin reduction may trigger downstream signalling.

Our demonstration that the isolated BAT-LOV* domain binds flavin and conformationally responds to chemical or photoreduction lends strength to the assertion that this module acts as an integrated redox/light sensor in the regulation of bacteriorhodopsin production. The reactivity of BAT-LOV* also raises the question as to whether ancestral LOV proteins were redox sensors that bound flavin but did not contain an adduct-forming Cys. Introduction of such a reactive Cys would preserve the light-adapted state and thereby increase effective light sensitivity. Increased photoreduction yields made possible by a neighbouring Cys donor, as we observe with BAT-LOV* P188C, may have been an intermediate step in the generation of an adduct mechanism. Structural changes that then promoted bond formation would have made photoreception less susceptible to changes in redox potential. As a result, Cys incorporation could have rapidly disseminated due to its utility for photosensing. It may be no accident that the flavin chromophores of certain LOV photoreceptors have redox potentials in the physiologically relevant range^46^. Thus, the division between photosensor and redox sensor may be small, particularly for flavoproteins in which the polypeptide responds to changes in the flavin redox state, whether they be generated chemically or by light.

## Methods

### Molecular biology and protein expression

VVD constructs were cloned into pET28a vectors and overexpressed in *E. coli* BL21(DE3) cells as previously described^11^,^12^. Expression of VVD variants was induced with 100 μM isopropyl β-D-1-thiogalactopyranoside (IPTG) for 20 h at 17 °C under constant light. Proteins were purified by Ni:NTA affinity chromatography, followed by SEC on HiLoad 26/60 Superdex 75 or 200 prep grade columns with 50 mM Hepes (pH 8), 150 mM NaCl, 10% (v/v) glycerol.

Site-directed mutants of YF1 were generated in the background of the expression plasmid pET-41a-YF1 (ref. 27) via the QuikChange protocol (Invitrogen, Life Technologies GmbH). For assaying YF1 activity *in vivo*, corresponding mutants were also introduced into the reporter plasmid pDusk-myc-DsRed^27^,^28^. Purification of YF1 WT and site-specific mutants was carried out as described previously^27^. Briefly, expression in *E. coli* BL21 CmpX13 cells^47^ was induced with 1 mM IPTG for 4 h at 37 °C. Proteins were purified by Ni:NTA affinity chromatography and dialysed into storage buffer (10 mM Tris-HCl (pH 8.0), 10 mM NaCl, 10% (v/v) glycerol). Protein concentration was determined by absorption measurements with an Agilent 8453 UV–vis spectrophotometer (Agilent Technologies, Santa Clara, CA, USA) using an extinction coefficient at 450 nm of 12,500 M^−1^ cm^−1^. Full-length FixJ was expressed and purified as previously^21^, with the exception that the N-terminal His_6_ affinity tag was not cleaved off. Protein concentration was calculated using an extinction coefficient of 4,860 M^−1^ cm^−1^ at 280 nm (ref. 21).

The gene from *Halorubrum hochstenium* (ATCC 700873) BAT, residues 141–275, was synthesized by Biomatik in Bluescript (pBSK+Simple), and was cloned into pET28a via restriction with NdeI and XhoI. The protein was overexpressed with an N-terminal His_6_ tag and purified from *E. coli* BL21(DE3) cells as described for VVD, except that after induction expression was executed at 37 °C for 3 h under constant light. After SEC, samples were further purified by HiPrep Q XL 16/10 to remove endogenous flavin. Samples were incubated with free flavin nucleotide in a buffer containing 0.5 M NaCl for at least 12 h. Unbound flavin was removed by buffer exchange through 10 kDa cutoff centrifugal filters (Amicon). Mutant variants of BAT were prepared by site-directed mutagenesis.

The identity of all constructs was confirmed by DNA sequencing at the Biotechnology Resource Center of Cornell University or by LGC Genomics (Berlin, Germany).

### Absorption and fluorescence spectroscopy

Photoreduced species of VVD and BAT were monitored by irradiating dark-state samples with 448-nm diode laser light (30 mW; World Star Tech) perpendicular to the observation beam. Full spectra were collected on an Agilent 8453 diode-array spectrophotometer as a function of time. In kinetics mode, data were obtained by monitoring samples at 450 nm with a cycle time of 0.5–1.0 s under temperature control. Traces were normalized and fit with MATLAB (The MathWorks Inc., Natick, MA, USA) to equation (1)


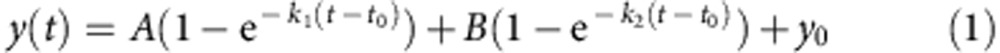


where *A*, *B*, *y*_0_ are coefficients, *t*_0_ is the *x*-axis offset, and *k*_1_ and *k*_2_ are rate constants. In the case of BAT-III and P188C, the data were fit to the triexponential version of equation (1).

Absorption spectra for YF1 variants were recorded with an Agilent 8453 spectrophotometer as described above except that samples were illuminated with 455-nm light (Royal Blue, Luxeon Star, 50 mW cm^−2^) at 22 °C until the photostationary state was reached. Photobleaching and recovery kinetics were followed by recording absorption spectra. Data evaluation was carried out with Origin (OriginLab, Northampton, MA, USA).

Fluorescence measurements were carried out on a Varian Cary Eclipse fluorometer. For kinetic measurements of fluorescent quenching by photoreduction, samples were excited at 450 nm using a 10-nm bandwidth, and emission data were collected at 508 nm using a 5-nm slit width with 0.1 s averaging. Kinetic traces were normalized and fit by MATLAB using equation (1) with *y*_0_=1. Fluorescence intensities used for relative quantum yield measurements were collected at 525 nm over the first 5 s of excitation at 450 nm. Reoxidation spectra of P188C were recorded by treatment with [Co(phen)_3_](ClO_4_)_3_˙2H_2_O, which was prepared as described^48^.

### Analytical SEC

Purified samples of VVD and BAT-LOV* were verified to be in their dark state by absorption spectroscopy, as indicated by the absence of adduct- or NSQ-related features. Samples were immediately loaded onto equilibrated foil-covered Superdex 75 or 200 10/300 GL columns. Light-activated samples were obtained by irradiation on ice until a significant amount of adduct or NSQ built up. Samples were checked by UV-vis spectroscopy and immediately loaded onto the uncovered column, with constant external illumination throughout the run. Reduced BAT samples were prepared anaerobically with the addition of 12 mM chromium (II) EDTA complex (Cr:EDTA)^49^ and immediately loaded onto the column with degassed buffer (50 mM Hepes (pH 7.5), 500 mM NaCl, 2 mM TCEP, 5 mM DTT). BAT-III samples were photoreduced in the same degassed buffer and loaded onto the column.

### Multi-angle light scattering

A 5.0 mg ml^−1^ solution of Bovine Serum Albumin (BSA, Sigma) was injected onto a Phenomenex Bio Sep-SEC-s 300 column that had been equilibrated in GF buffer containing 50 mM Tris (pH 7.5) and 150 mM NaCl to normalize the light-scattering detectors and act as a calibration control for both peak alignment and molecular weight determinations. Purified protein samples (1–10 mg ml^−1^) were then injected onto the same column. BAT-III samples were run using degassed buffer (50 mM Hepes (pH 7.5), 500 mM NaCl, 2 mM TCEP, 5 mM DTT). The SEC (WTC050N5—Wyatt) is coupled to a static 18-angle light-scattering detector (DAWN HELEOS-II), a refractive index detector (Optilab T-rEX; Wyatt Technology) and dynamic light-scattering device (WyattQELS). Data were collected every second for 30 min at the flow rate of 1 ml min^−1^ at 25 °C. The ASTRA V software was used to extract the molar weight distribution, root-mean-square radius, radius of hydration and the polydispersity of each resolved peak, which were taken as averages across the elution peaks. Concentrations were determined by the refractive index indicator.

### YF1 *in vivo* and *in vitro* activity assays

*In vivo* activity measurements of YF1 WT and variants were conducted in the pDusk-DsRed reporter system as described^28^. Briefly, for each construct, three 5-ml LB/Kan cultures were incubated overnight at 37 °C and 225 r.p.m. either in the dark or under constant blue light (470 nm, 100 μW cm^−2^). OD_600_ and DsRed fluorescence were measured using black-walled 96-well μClear plates (Greiner BioOne, Frickenhausen, Germany) with a Tecan Infinite M200 PRO plate reader (Tecan Group Ltd. Mannedorf, Switzerland). Fluorescence excitation and emission wavelengths were set at 554±9 nm and 591±20 nm, respectively. Data were normalized to the fluorescence per OD_600_ for YF1 WT under dark conditions and represent the averages of three biological replicates±s.d. Light-dose experiments were conducted as above except that the intensity of 470-nm light was varied between 0 and 150 μW cm^−2^. Light intensities were determined with a power meter (model 842-PE, Newport) and a silicon photodetector (model 918D-UV-OD3, Newport).

Net kinase activities of YF1 variants were assessed *in vitro* by monitoring the binding of phospho-FixJ to DNA using EMSA. A double-stranded DNA fragment containing part of the FixK2 promoter sequence was produced by heating to 95 °C and then slowly cooling a mixture of 100 μM forward oligonucleotide primer (5′-GAGCGATATCTTAAGGGGGGTGCCTTACGTAGAACCC-3′) labelled at its 5′-end with (5-and-6)-carboxytetramethylrhodamine (TAMRA) and 100 μM of the reverse complementary primer in 5 mM Tris-HCl (pH 8.0); the high-affinity FixJ binding site is underlined^50^,^51^. To analyse net kinase activities, 250 nM YF1 WT or variants were mixed with 1 mM ATP and 1.25 μM FixK2-DNA substrate in 10 mM HEPES (pH 8.0), 80 mM KCl, 2.5 mM MgCl_2_, 0.1 mM EDTA, 100 μg ml^−1^ BSA, 10% (v/v) glycerol, 4% (v/v) ethylene glycol and 10 mM Tris(2-carboxyethyl)phosphine. In case of YF1 variants harbouring the mutation C62A, a ROS scavenger system containing 0.5 mg ml^−1^ glucose oxidase, 5 mg ml^−1^ glucose and 30 U catalase was added to the reaction mixture, and the buffer concentration was raised to 200 mM HEPES. The mixture was either kept in the dark or incubated under constant blue light (455 nm, 50 mW cm^−2^) at 30 °C for 15 min followed by addition of 30 μM FixJ. Samples were further incubated for 30 min and then run on a native 6% (w/v) acrylamide gel in TBE buffer (89 mM Tris, 89 mM borate, 2 mM ethylenediaminetetraacetic acid, pH 8.3) at 100 V for 45 min. DNA bands were visualized in a Fujifilm image reader FLA 3000 (Fujifilm Holdings K. K.) using excitation and emission wavelengths of 532 and 580 nm, respectively.

### Electron spin resonance spectroscopy

For pulsed-dipolar ESR experiments, samples containing 350 μM VVD and 30% glycerol (v/v) were irradiated with 448-nm diode laser light on ice for several minutes until uniformly pale blue in colour and then were flash-frozen in liquid nitrogen. Four-pulse DEER experiments were conducted at 100 K on a 17.3 GHz Fourier Transform ESR spectrometer, which is modified to perform pulsed-dipolar ESR experiments^52^,^53^. π/2 and π pulses were 20 ns and 40 ns, respectively, with a frequency separation of 60 MHz. Pumping was applied at the high-field side of the ESR spectrum, with detection on the opposite low-field slope. Data averaging time was 23 h. The baseline used for data processing was approximated by a single exponential function, which slightly deviates from a linear polynomial. Distance distributions of spin separations within the sample were calculated by the Tikhonov method and refined by the maximum entropy regularization^53^.

For cw-ESR measurements on YF1 variants, *E. coli* cultures were transferred into quartz tubes (QSIL GmbH, Ilmenau, Germany; 3.0 mm/3.9 mm inner/outer diameter), were either kept in the dark or were illuminated with a 450 nm LED (LUXEON Lumiled, Phillips Lumileds, San Jose, CA, USA) for 5 min, and were then rapidly frozen in liquid nitrogen. cw-ESR spectra were recorded on a laboratory-built X-Band spectrometer, consisting of a microwave bridge ER 041 MR, microwave controller ER 048R, magnet power supply ER 081S, field controller BH 15 and cavity resonator ER 4122 SHQ E all from Bruker. For signal detection, a Stanford Research SR810 lock-in detector (Stanford Research, Sunnyvale, CA, USA) was used. Microwave frequency measurements were performed using an Agilent 53181A frequency counter (Agilent Technologies). The samples were measured with 4G modulation amplitude, 100 kHz modulation frequency and 100 ms lock-in time constant. The microwave power was 60 μW and the frequency was ∼9.38 GHz. For each measurement, the current microwave frequency was recorded. The spectra were then normalized to 9.6 GHz. During cw-ESR measurements, samples were maintained at 80 K with an Oxford ESR 910 cryostat and Oxford ITC503 temperature controller. The reported spectra are averages over 40 scans.

### Sequence analysis and homology modelling

Using Biopython, a BLAST search was performed with the residues 1–127 of *B. subtilis* YtvA (PHOT_BACSU) as the query sequence and with an *E*-value of 10 as cutoff. The BLAST results were filtered for entries that lack the adduct-forming cysteine (corresponding to residue C62 in YtvA) but possess at least 9 out of the other 10 conserved amino acids in LOV domains (corresponding to residues G59, N61, R63, F64, L65, Q66, N94, N104 and Q123 in YtvA). The resultant list was manually curated to remove entries that correspond to proteins in which the active-site cysteine was deliberately removed by mutation, for example, in LOV domains used as fluorophores. Sequences were further filtered to remove closely similar entries (cutoff 90% sequence identity). All remaining entries were aligned to the sequences of VVD and YtvA using ClustalX^54^.

A homology model of BAT-LOV* was calculated using SWISS-MODEL^55^ and YtvA as the template structure (PDB entry 2PR6), which possesses a sequence similarity of 45.2% compared with BAT-LOV*. Sequence alignment was achieved with Clustal Omega at EMBL-EBI^54^.

## Additional information

**How to cite this article:** Yee, E. F. *et al.* Signal transduction in light–oxygen–voltage receptors lacking the adduct-forming cysteine residue. *Nat. Commun.* 6:10079 doi: 10.1038/ncomms10079 (2015).

## Supplementary Material

Supplementary Figures Supplementary Figures 1-9

Supplementary Data 1 Overview of LOV-Like Proteins Lacking the Adduct-Forming Cysteine. The accompanying HTML File can be opened in a standard web browser and provides a graphical overview of the domain architecture of entries in GenBank that comprise LOV-Like domains lacking the adduct-forming cysteine residue.

## Figures and Tables

**Table 1:** Relative quantum yield of fluorescence for LOV variants.

VVD-III	≡1.00
BAT-LOV*	0.08±0.02
BAT-LOV* N252C	0.10±0.02
BAT-II Y163F:Y247F	0.08±0.03
BAT-III Y163F:Y247F:W172F	0.58±0.13

BAT, bacterio-opsin activator; LOV, light–oxygen–voltage; VVD, vivid.
