# Supplementary material for: Signal transduction in light–oxygen–voltage receptors lacking the adduct-forming cysteine residue
Source: Nat Commun. 2015 Dec 9;6:10079. doi: 10.1038/ncomms10079 (PMC4682037; doi:10.1038/ncomms10079)
Supplement: Supplementary Figures — 1-9 [file ncomms10079-s1.pdf]

Supplementary Information

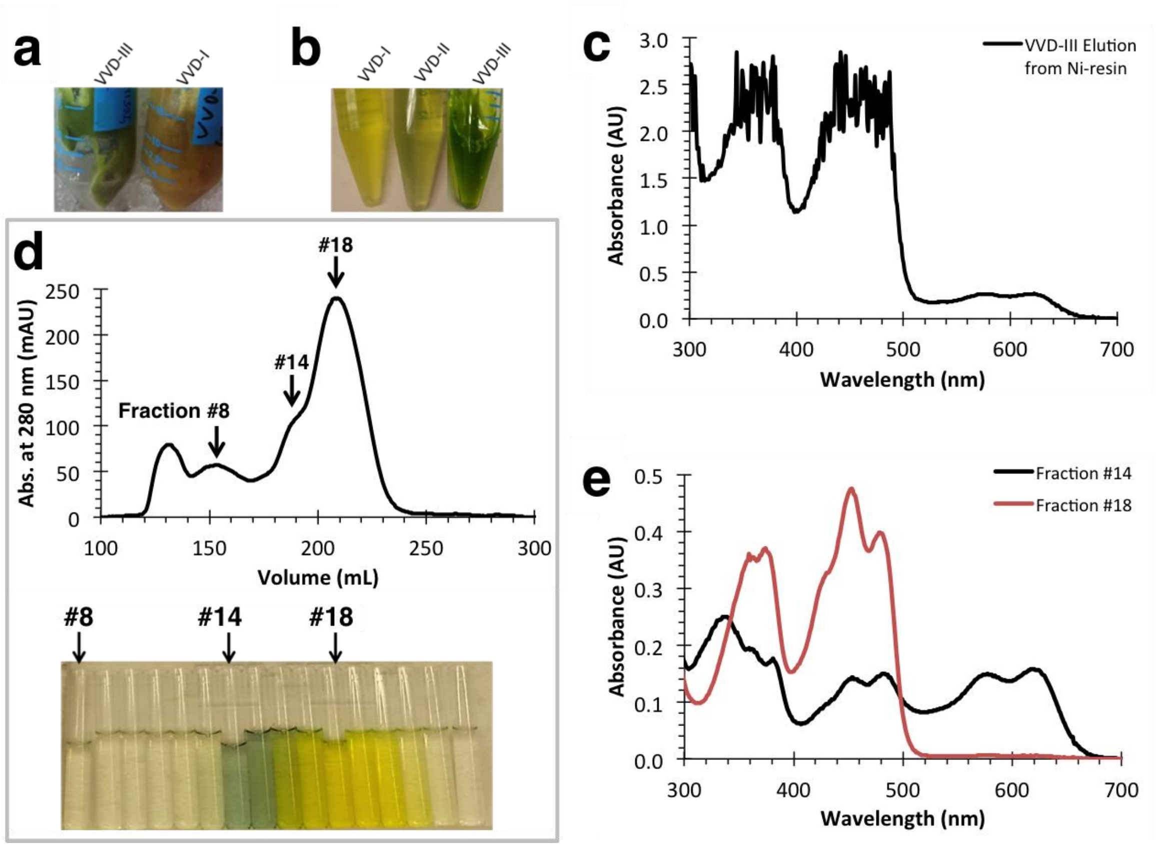

Supplemental Figure 1. VVD-III purifies in a reduced state. (**a**) The cell pellet of VVD-III (VVD Δ36 C108A:M135I:M165I) is green compared to VVD-I (wild type VVDΔ36) due to the accumulation of the neutral semiquinone radical. (**b**) Purified VVD-I is yellow, VVD-II (VVD Δ36 M135I:M165I) is pale green, while VVD-III is dark green. (**c**) Absorption spectrum of purified VVD-III, shown in (**b**), reveals a mixture of oxidized (450 nm) and neutral semiquinone (577 nm, 621 nm) states of FAD. (**d**) Size Exclusion Chromatography (SEC) profile of VVD-III shows that the different redox forms of VVD separate by size; fractions corresponding to the peaks are shown above. (**e**) Absorption spectra of fraction #14 (**d**), which elutes earlier than #18, contains a neutral semiquinone state of VVD-III, whereas #18 contains the oxidized form.

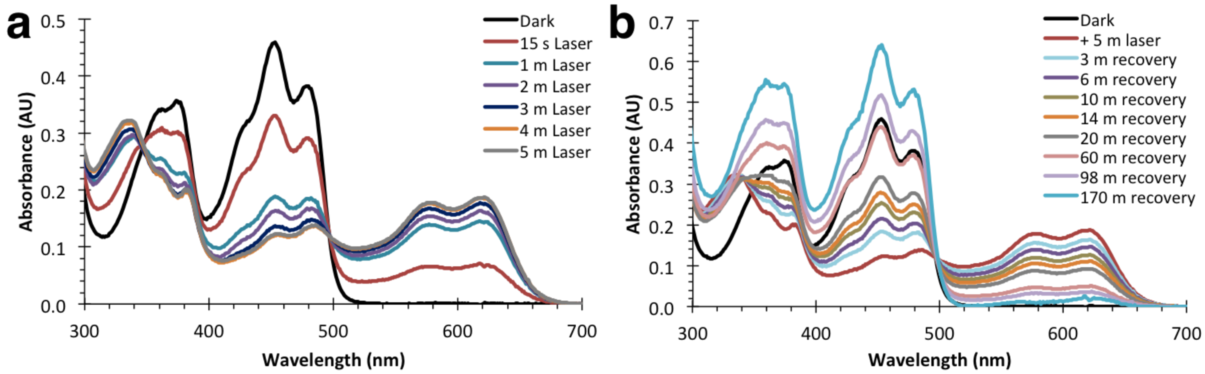

Supplementary Figure 2. Photoreduction of VVD-III. (**a**) Time course for photoreduction of VVD-III to a neutral semiquinone with a 30 mW 448-nm laser, as monitored by absorption spectroscopy (note the two isosbestic points at 347 nm and 497 nm). (**b**) Reoxidation of VVD-III under ambient aerobic conditions. Precipitation of some protein during the experiments causes light scattering and greater absorption at wavelengths <500 nm compared to the dark-adapted sample.

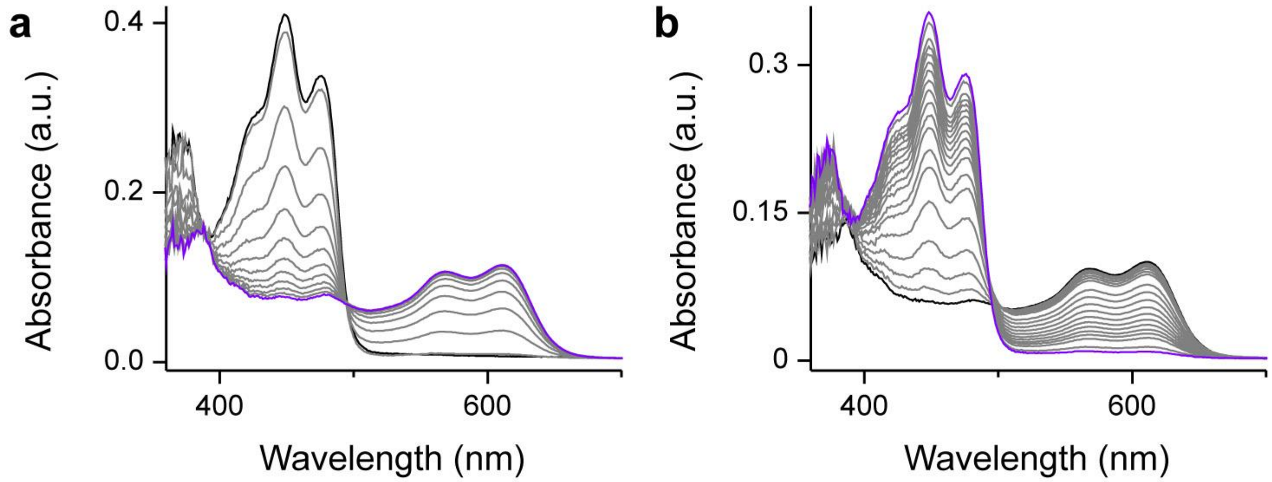

Supplementary Figure 3. Photoreduction of YF1 C62A.

(a) Photoreduction of YF1 C62A in the presence of 10 mM TCEP was followed by absorption spectroscopy. The sample was continuously illuminated with 455-nm light (50 mW cm^{-2}), and absorption spectra were recorded using a diode-array spectrophotometer. To avoid excitation of the sample by probe light, spectra were recorded with the tungsten lamp only, with the deuterium lamp turned off; hence spectra below ~370 nm suffer from poor signal-to-noise ratio. From top to bottom, spectra were recorded 1, 62, 122, 182, 242, 302, 362, 422, 482, 542, 602, and 662 s after onset of illumination.

(b) Following saturating photoreduction to the NSQ state, the recovery was monitored in the dark under otherwise identical experimental settings. From top to bottom, spectra were recorded 0, 120, 240, 390, 570, 750, 930, 1440, 1800, 2160, 2520, 2880, 3240, 3600, 3960, 4320, 4750, 6190, and 7630 s after blue-light illumination ceased.

a

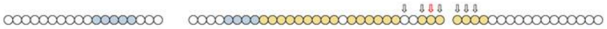

```
NcVivid       63 ELGPVDTSCALILCDLKQ---KDTPIVYASEAFLYMTGYSNAEVLGRNCR-FLQSPDGMVKPKSTRKY 126
BsYtvA        17 KKALDHVRVGVVITDPAL---EDNPIVYVNQGFVQMTGYETEEILGKNCR-FLQGKH-----------  69
BAT-LOV*     143 TRAVEDAPIGISISDPDL---PDYPLVYVNDAWREHTGYSVEEVLGRNPR-FLQGPG----------- 195
AzNifL        28 RQTVEHAPIAISITDLKA------NILYANRAFRTITGYGSEEVLGKNES-ILSNGT-----------  77
EcAer         11 NTPLADDTTLMSTTDLQS------YITHANDTFVQVSGYTLQELQGQPHN-MVRHPD-----------  60
504822023     11 REALNHTNVGLIITDPNQ---DHHPIIFANKGYANLTGYEIDEVIGKNGR-LLQGEE-----------  63
494809730     12 YKALRLSPSPVIIADPTI---EGTPFVFVNHSFEKLTGYSKDEIIGKNGS-FLQGPD-----------  64
655089558     22 FQAIDRMGVGLAITNPNL---PDNPLVYVNQGFEKITGYKREEVLHRNLR-FLQGKE-----------  74
675493957     21 LRAIDLMGVGLAITDPRL---QDNPLVYVNRGFEEITGYSREEVLLRNPR-FLQGAE-----------  73
682411003    477 -LGSVDMSCAFVVCDVTM---PDYPIIYASEIFSRLTGYNKNEVWMENSR-FLQSPDGKTQKGEKRKY 539
449453764    416 ATTLERIKKIFFITNPRL---PDNPIIFASHRFLDSTEYTLEEVLGRNFC-FLQGPE----------- 468
457693        43 ATTLERIEKNFVITDPRL---PDNPIIFASDSFLELTEFSRAEILARNRR-FLQGPE-----------  95
148907121     35 REALGQVHFNFVITDPCL---LDHPIVYASQGFLKMSGYSTEEVVGRNVR-FLQGPD-----------  87
224110804    239 NISLGRINQSFVLIDPHL---PNMPIVYASDAFLKLTGYDRHEVLGCNWR-FLSGVG----------- 291
367045084    271 --PYHGLGDAFCLSDPLL---PDNPIIHASDGLLAMSGFRRKELVGKNGR-VLQGVA----------- 321
302887177    293 RDISEGLTEVFYLSDPSR---PDNPIVFASEEFHRTTQYGMNYAIGRNYR-FLQGPK----------- 345
675353822    143 -LQADVTGLSFLLTDPRL---PGGPIVYASPGFLALTGHGAGDVLGRNPR-LLQGRE----------- 194
635148931     59 RDALTAFQQSFCMVDATK---PDLPVLFASEGFYQLTGYSALETIGKNPR-FLQGQE----------- 111
635148707    612 KDALGSFPHAFVVCDAGK---PDYPIIFASAGFFGMTGYGTKEVLGANPR-FLQGPD----------- 664
298708645    539 KTMSETVSVGMVVADMNV---PGCPLAYINEGFKTVTGYG-KENIGRNSK-FLQGEE----------- 590
495708415     19 -RLGVKFHTSFLVINSEV---EDQPIVYANDAFFKMIGYSEEETLGRNGR-FLHGEK-----------  70
493883015     19 -RLGVEFHTSFLVINSEM---EDQPVVYANAAFYKMIGYSEAETLGRNGR-FLHGEK-----------  70
552171632    186 QRGIEASPHGILMADAQQ---PDHPVVYVNPAFCEITGYPREEVLGQNSR-FLQGSD----------- 238
517214330    303 KRGIEASPTGMLIVDARS---PDMPTVYANLAYADMTGYTQNDLIGHNRR-FLHGDD----------- 355
515954762    299 KRGVDASPSGMFITDAS----ENMVISFVNPAFLKQVGYTEEEVIGRNYN-FLQGRD----------- 350
491039211    302 ERAIQNSTHGVVIAHAD----ADMPIVYVNAAFLAMTGYTESEIVGRNWR-FLKGPD----------- 353
548767960    316 ESVITNSTDSVIITESNLLDAPGPKIIYVNNAFTRLTGYGSEEVMGRNPR-FLQGPN----------- 371
496124663    143 KRAVAEAPVGVSISDPDL---PDYPLVYANEAWVDHTGYPIEEALGRNPR-FLQGPG----------- 195
558583704     58 ERAVNEAPVGLSLCDPDL---PDYPLVYVNGAWERHTGYSSEEVLGRNPR-FLQGPE----------- 110
544602271    141 QRAIEAAPIGVSLTDPEL---SDNPLVYVNDTWGEFTGYDSEEMLGRNPR-LLQGVG----------- 193
493724709    138 VRAIEDAPIGISLSDPSL---PDNPLVYVNRAWEELTGYEEETVLGRNPR-LLQGPD----------- 190
491747656    138 VRAIDDAPIGISLSDPSL---PDNPTVYVNDAWEEITGFDRASILGRNPR-ILQGPA----------- 190
492946794    300 NRAMDEAQVGISISDIRE---PNEPLIYVNEGFERVTGYSAEETLGRNLC-FLQGEQ----------- 352
505222758    655 EQAIDSSNVGVIITDPQR---EDNPIEYINKGFSDITGYQEADVVGCNPR-FLHGPE----------- 707
499544181    657 NRAMDEATVGIQITDPTQ---ADNPLVYVNDGFERMTGYTAEDALGRNPR-FLQGAD----------- 709
496124150    202 ERALDEAGTSIQITDPTQ---PGNPLVYVNEEFVQLTGYERSEALGRNPR-FLQGPG----------- 254
491108658    372 RRAIDEASVGITITGPSE---DDCPIVYANAAFEELTGYDTDEILGENHR-VLQGPD----------- 424
494345671    262 NRAIDEAPVGITIADANR---PDNPLVYANDKFSELTGYQEENIVGQNSR-FLQGEN----------- 314
517068699     68 LWLLDDAPVGLTLSGPAY---QDNPIQYVNRTFRDLTGYSLEEVLDENPR-FLQGPD----------- 120
558583472     63 LWVLGAAPFGLTLAGPAY---QDTPIVYANRTFRRLTGYPLSAVRGENLR-LLQGPE----------- 115
505221353     68 LWVLGDASVGLTLAGPAF---QDTPTIYANRTFRAVTGYSLSAVRGENLR-LLQVPE----------- 120
495587172     70 MVRLGTAPMGLTLTGPAY---QDNPLRYATQTFREMSGYSLTALRGENLR-LLQGPA----------- 122
493940260     71 AWMLAEAPLGVVVAGAAY---HDNPVLYANRATRRLTGHSLAALWGENLR-RLQGPG----------- 123
541203563     74 AWVLDHAPVGVTLTGAAY---HDNPLQYANLAFRELTGHDTTRLSGANPR-LLQTET----------- 126
544601395     59 TQAMDDTPVSMVISGPAY---TDNPIWYANDAFERLTGYDEREVLGRNLR-LLQGPE----------- 111
499205526    161 EQALDEAPIGITISDATD---PEEPIIYINDSFEDITGYSPDEVVGANHR-FLQGPK----------- 213
652397765    821 ERAIAYSSNGIVIADARK---PDNPIVFVNPAFEKVTGYSAAEALGKNSR-FLQGKD----------- 873
497470070    755 ERAIAASNNGIVLADARL---EDHPIIFVNPAFEKITGYSASEIIGENTR-FLQGTD----------- 807
516324372    587 ERAIAASSNGITITDAQL---PDTPVVYVNPSFEKLTGYSAAEAIGRNLR-FLQGSD----------- 639
516330201    790 DRAMAASSNGIIISDARQ---PDLPLIYVNSAFERITGYSAAEVIGQNSR-FLQGDE----------- 842
497074813    553 DRAIAASSNGIVICDARL---PNLPIIYANPAFEYITGYSPEEVIGRNFR-FLQGND----------- 605
515878998    549 ERAIEASNNGIVISDARL---PNMPIIYTNPAFEHITGYCRDDVIGRNSS-FLQGID----------- 601
505013918    539 DRALAASTNGIIITDARL---PNLPIVYVNQSFEKITGYSACEVIGRNSR-FLQKSD----------- 591
499697421    683 LRAIESTGNSIIIADAQQ---PDMPIIYVNPAFEKITGFSAAEVIGRNFR-FLQGLE----------- 735
517848989    370 QRSIEASYNGIAIADAQA---PDMPIIYVNSSFERITGYRAAEAIGKNSR-FLQGAD----------- 422
652892586    500 ERAVEASASSISMVDVNR---PGMPLIYVNPAFERITGYSRDEAIGRNAR-FLQGKE----------- 552
648454791    293 QRAIDASPMGVCIADARQ---PDLPLIYCNPAFERMTGYSRDEVIGRNGR-FLQGVD----------- 345
489552830    299 NLALRASPLGVTIADARQ---PEQPLIYCNPAFERMSGYSRDEALGNNWR-FLLHHD----------- 351
639204164    632 RRVVESVPSGITVADAQQ---PDFPIVYVNPGFERMTGYRAEEILGRNAR-FLHSSE----------- 684
499703955    331 DMVLSNVSNGVTIAEVGR---GDLPLIYANPAFEHITGYLAKNVLGRNAR-FLHEFD----------- 383
568205117    348 EQVLQTVSNGVAIADATQ---PDFPLVYVNPAFESMTGYKAADVVGTNAR-FLHRDD----------- 400
491515482    165 DRAIEASSAGIIITDAKR---KNHPIIYANPAFSQMTGYSREELIGLNPR-ILQGPE----------- 217
374852440    381 DRAIEASSVGILITDALK---PDNPIIYANPAFLRITGYSLEELLGKNPR-FLQGPE----------- 433
504991123    209 DRAIAATSTGVTISDATD---PDNPIIYCNPAFESMTGYRRQEIIGKNGR-FLHGSD----------- 261
504955604    566 DRAIAAASFGLCIADATQ---PDYPIVYCNPAFEKMTGYSHSEAIGRNYR-FLQGAD----------- 618
495504198    257 DLAIAEVDTGILITDARE---DDNPIVYANKAMEQMTGYTMQEMLGQNPR-FLQGDD----------- 309
503059604     13 AQAVDQLHDGITIADARQ---QDWPLIYVNAGFEKLSGYSAAELIGKPVR-FLQGTD-----------  65
251772029    924 SSAVAAVGEGVSISDLDG------KILFVNESFTTITGYSQEEVQGKNLR-ILQGEG----------- 973
651235784    233 TSAIQSTHLGLVVTDQKR------RVTWCNKGFEEMSEYSLREVKGQNLSGFLQGED----------- 283
```

```
NcVivid      63 ELGPVDTSCALILCDLKQ---KDTPIVYASEAFLYMTGYSNAEVLGRNCR-FLQSPDGMVKPKSTRKY 126
BsYtvA       17 KKALDHVRVGVVITDPAL---EDNPIVYVNQGFVQMTGYETEEILGKNCR-FLQGKH----------  69
BAT-LOV*    143 TRAVEDAPIGISISDPDL---PDYPLVYVNDAWREHTGYSVEEVLGRNPR-FLQGPG---------- 195
AzNifL       28 RQTVEHAPIAISITDLKA------NILYANRAFRTITGYGSEEVLGKNES-ILSNGT----------  77
EcAer        11 NTPLADDTTLMSTTDLQS------YITHANDTFVQVSGYTLQELQGQPHN-MVRHPD----------  60
504822023    11 REALNHTNVGLIITDPNQ---DHHPIIFANKGYANLTGYEIDEVIGKNGR-LLQGEE----------  63
494809730    12 YKALRLSPSPVIIADPTI---EGTPFVFVNHSFEKLTGYSKDEIIGKNGS-FLQGPD----------  64
655089558    22 FQAIDRMGVGLAITNPNL---PDNPLVYVNQGFEKITGYKREEVLHRNLR-FLQGKE----------  74
675493957    21 LRAIDLMGVGLAITDPRL---QDNPLVYVNRGFEEITGYSREEVLLRNPR-FLQGAE----------  73
682411003   477 -LGSVDMSCAFVVCDVTM---PDYPIIYASEIFSRLTGYNKNEVWMENSR-FLQSPDGKTQKGEKRKY 539
449453764   416 ATTLERIKKIFFITNPRL---PDNPIIFASHRFLDSTEYTLEEVLGRNFC-FLQGPE---------- 468
457693       43 ATTLERIEKNFVITDPRL---PDNPIIFASDSFLELTEFSRAEILARNRR-FLQGPE----------  95
148907121    35 REALGQVHFNFVITDPCL---LDHPIVYASQGFLKMSGYSTEEVVGRNVR-FLQGPD----------  87
224110804   239 NISLGRINQSFVLIDPHL---PNMPIVYASDAFLKLTGYDRHEVLGCNWR-FLSGVG---------- 291
367045084   271 --PYHGLGDAFCLSDPLL---PDNPIIHASDGLLAMSGFRRKELVGKNGR-VLQGVA---------- 321
302887177   293 RDISEGLTEVFYLSDPSR---PDNPIVFASEEFHRTTQYGMNYAIGRNYR-FLQGPK---------- 345
675353822   143 -LQADVTGLSFLLTDPRL---PGGPIVYASPGFLALTGHGAGDVLGRNPR-LLQGRE---------- 194
635148931    59 RDALTAFQQSFCMVDATK---PDLPVLFASEGFYQLTGYSALETIGKNPR-FLQGQE---------- 111
635148707   612 KDALGSFPHAFVVCDAGK---PDYPIIFASAGFFGMTGYGTKEVLGANPR-FLQGPD---------- 664
298708645   539 KTMSETVSVGMVVADMNV---PGCPLAYINEGFKTVTGYG-KENIGRNSK-FLQGEE---------- 590
495708415    19 -RLGVKFHTSFLVINSEV---EDQPIVYANDAFFKMIGYSEEETLGRNGR-FLHGEK----------  70
493883015    19 -RLGVEFHTSFLVINSEM---EDQPVVYANAAFYKMIGYSEAETLGRNGR-FLHGEK----------  70
552171632   186 QRGIEASPHGILMADAQQ---PDHPVVYVNPAFCEITGYPREEVLGQNSR-FLQGSD---------- 238
517214330   303 KRGIEASPTGMLIVDARS---PDMPTVYANLAYADMTGYTQNDLIGHNRR-FLHGDD---------- 355
515954762   299 KRGVDASPSGMFITDAS----ENMVISFVNPAFLKQVGYTEEEVIGRNYN-FLQGRD---------- 350
491039211   302 ERAIQNSTHGVVIAHAD----ADMPIVYVNAAFLAMTGYTESEIVGRNWR-FLKGPD---------- 353
548767960   316 ESVITNSTDSVIITESNLLDAPGPKIIYVNNAFTRLTGYGSEEVMGRNPR-FLQGPN---------- 371
496124663   143 KRAVAEAPVGVSISDPDL---PDYPLVYANEAWVDHTGYPIEEALGRNPR-FLQGPG---------- 195
558583704    58 ERAVNEAPVGLSLCDPDL---PDYPLVYVNGAWERHTGYSSEEVLGRNPR-FLQGPE---------- 110
544602271   141 QRAIEAAPIGVSLTDPEL---SDNPLVYVNDTWGEFTGYDSEEMLGRNPR-LLQGVG---------- 193
493724709   138 VRAIEDAPIGISLSDPSL---PDNPLVYVNRAWEELTGYEEETVLGRNPR-LLQGPD---------- 190
491747656   138 VRAIDDAPIGISLSDPSL---PDNPTVYVNDAWEEITGFDRASILGRNPR-ILQGPA---------- 190
492946794   300 NRAMDEAQVGISISDIRE---PNEPLIYVNEGFERVTGYSAEETLGRNLC-FLQGEQ---------- 352
505222758   655 EQAIDSSNVGVIITDPQR---EDNPIEYINKGFSDITGYQEADVVGCNPR-FLHGPE---------- 707
499544181   657 NRAMDEATVGIQITDPTQ---ADNPLVYVNDGFERMTGYTAEDALGRNPR-FLQGAD---------- 709
496124150   202 ERALDEAGTSIQITDPTQ---PGNPLVYVNEEFVQLTGYERSEALGRNPR-FLQGPG---------- 254
491108658   372 RRAIDEASVGITITGPSE---DDCPIVYANAAFEELTGYDTDEILGENHR-VLQGPD---------- 424
494345671   262 NRAIDEAPVGITIADANR---PDNPLVYANDKFSELTGYQEENIVGQNSR-FLQGEN---------- 314
517068699    68 LWLLDDAPVGLTLSGPAY---QDNPIQYVNRTFRDLTGYSLEEVLDENPR-FLQGPD---------- 120
558583472    63 LWVLGAAPFGLTLAGPAY---QDTPIVYANRTFRRLTGYPLSAVRGENLR-LLQGPE---------- 115
505221353    68 LWVLGDASVGLTLAGPAF---QDTPTIYANRTFRAVTGYSLSAVRGENLR-LLQVPE---------- 120
495587172    70 MVRLGTAPMGLTLTGPAY---QDNPLRYATQTFREMSGYSLTALRGENLR-LLQGPA---------- 122
493940260    71 AWMLAEAPLGVVVAGAAY---HDNPVLYANRATRRLTGHSLAALWGENLR-RLQGPG---------- 123
541203563    74 AWVLDHAPVGVTLTGAAY---HDNPLQYANLAFRELTGHDTTRLSGANPR-LLQTET---------- 126
544601395    59 TQAMDDTPVSMVISGPAY---TDNPIWYANDAFERLTGYDEREVLGRNLR-LLQGPE---------- 111
499205526   161 EQALDEAPIGITISDATD---PEEPIIYINDSFEDITGYSPDEVVGANHR-FLQGPK---------- 213
652397765   821 ERAIAYSSNGIVIADARK---PDNPIVFVNPAFEKVTGYSAAEALGKNSR-FLQGKD---------- 873
497470070   755 ERAIAASNNGIVLADARL---EDHPIIFVNPAFEKITGYSASEIIGENTR-FLQGTD---------- 807
516324372   587 ERAIAASSNGITITDAQL---PDTPVVYVNPSFEKLTGYSAAEAIGRNLR-FLQGSD---------- 639
516330201   790 DRAMAASSNGIIISDARQ---PDLPLIYVNSAFERITGYSAAEVIGQNSR-FLQGDE---------- 842
497074813   553 DRAIAASSNGIVICDARL---PNLPIIYANPAFEYITGYSPEEVIGRNFR-FLQGND---------- 605
515878998   549 ERAIEASNNGIVISDARL---PNMPIIYTNPAFEHITGYCRDDVIGRNSS-FLQGID---------- 601
505013918   539 DRALAASTNGIIITDARL---PNLPIVYVNQSFEKITGYSACEVIGRNSR-FLQKSD---------- 591
499697421   683 LRAIESTGNSIIIADAQQ---PDMPIIYVNPAFEKITGFSAAEVIGRNFR-FLQGLE---------- 735
517848989   370 QRSIEASYNGIAIADAQA---PDMPIIYVNSSFERITGYRAAEAIGKNSR-FLQGAD---------- 422
652892586   500 ERAVEASASSISMVDVNR---PGMPLIYVNPAFERITGYSRDEAIGRNAR-FLQGKE---------- 552
648454791   293 QRAIDASPMGVCIADARQ---PDLPLIYCNPAFERMTGYSRDEVIGRNGR-FLQGVD---------- 345
489552830   299 NLALRASPLGVTIADARQ---PEQPLIYCNPAFERMSGYSRDEALGNNWR-FLLHHD---------- 351
639204164   632 RRVVESVPSGITVADAQQ---PDFPIVYVNPGFERMTGYRAEEILGRNAR-FLHSSE---------- 684
499703955   331 DMVLSNVSNGVTIAEVGR---GDLPLIYANPAFEHITGYLAKNVLGRNAR-FLHEFD---------- 383
568205117   348 EQVLQTVSNGVAIADATQ---PDFPLVYVNPAFESMTGYKAADVVGTNAR-FLHRDD---------- 400
491515482   165 DRAIEASSAGIIITDAKR---KNHPIIYANPAFSQMTGYSREELIGLNPR-ILQGPE---------- 217
374852440   381 DRAIEASSVGILITDALK---PDNPIIYANPAFLRITGYSLEELLGKNPR-FLQGPE---------- 433
504991123   209 DRAIAATSTGVTISDATD---PDNPIIYCNPAFESMTGYRRQEIIGKNGR-FLHGSD---------- 261
504955604   566 DRAIAAASFGLCIADATQ---PDYPIVYCNPAFEKMTGYSHSEAIGRNYR-FLQGAD---------- 618
495504198   257 DLAIAEVDTGILITDARE---DDNPIVYANKAMEQMTGYTMQEMLGQNPR-FLQGDD---------- 309
503059604    13 AQAVDQLHDGITIADARQ---QDWPLIYVNAGFEKLSGYSAAELIGKPVR-FLQGTD----------  65
251772029   924 SSAVAAVGEGVSISDLDG------KILFVNESFTTITGYSQEEVQGKNLR-ILQGEG---------- 973
651235784   233 TSAIQSTHLGLVVTDQKR------RVTWCNKGFEEMSEYSLREVKGQNLSGFLQGED---------- 283
```

b

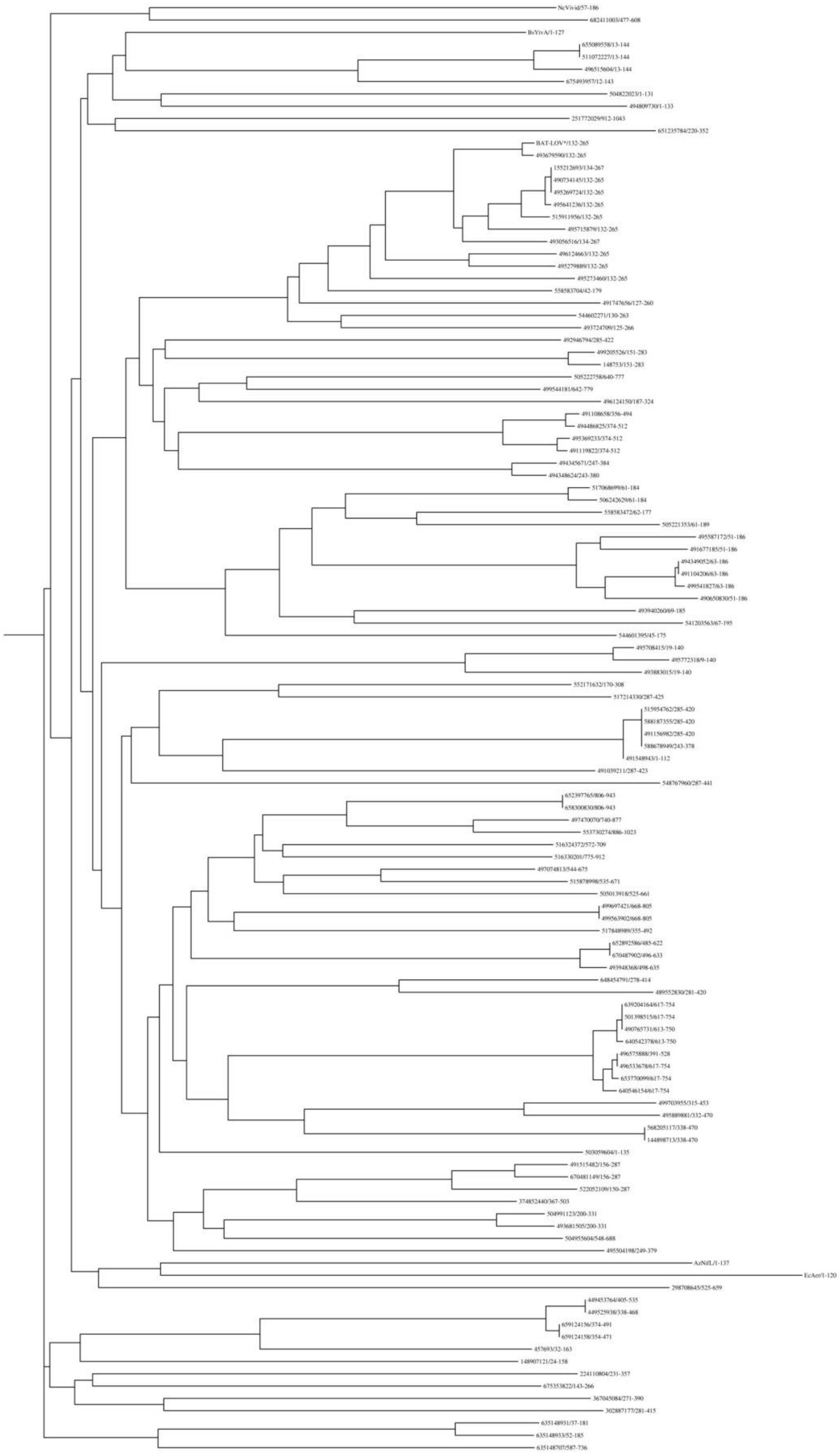
NcVivid/57-186
682411003/477-608
BsYtvA/1-127
655089558/13-144
511072227/13-144
496515604/13-144
675493957/12-143
504822023/1-131
494809730/1-133
251772029/912-1043
651235784/220-352
BAT-LOV*/132-265
493679590/132-265
155212693/134-267
490734145/132-265
495269724/132-265
495641236/132-265
515911956/132-265
495715879/132-265
493056516/134-267
496124663/132-265
495279889/132-265
495273460/132-265
558583704/42-179
491747656/127-260
544602271/130-263
493724709/125-266
492946794/285-422
499205526/151-283
148753/151-283
505222758/640-777
499544181/642-779
496124150/187-324
491108658/356-494
494486825/374-512
495369233/374-512
491119822/374-512
494345671/247-384
494348624/243-380
517068699/61-184
506242629/61-184
558583472/62-177
505221353/61-189
495587172/51-186
491677185/51-186
494349052/63-186
491104206/63-186
499541827/63-186
490650830/51-186
493940260/69-185
541203563/67-195
544601395/45-175
495708415/19-140
495772318/9-140
493883015/19-140
552171632/170-308
517214330/287-425
515954762/285-420
588187355/285-420
491156982/285-420
588678949/243-378
491548943/1-112
491039211/287-423
548767960/287-441
652397765/806-943
658300830/806-943
497470070/740-877
553730274/886-1023
516324372/572-709
516330201/775-912
497074813/544-675
515878998/535-671
505013918/525-661
499697421/668-805
499563902/668-805
517848989/355-492
652892586/485-622
670487902/496-633
493948368/498-635
648454791/278-414
489552830/281-420
639204164/617-754
501398515/617-754
490765731/613-750
640542378/613-750
496575888/391-528
496533678/617-754
653770099/617-754
640546154/617-754
499703955/315-453
495889881/332-470
568205117/338-470
144898713/338-470
503059604/1-135
491515482/156-287
670481149/156-287
522052109/150-287
374852440/367-503
504991123/200-331
493681505/200-331
504955604/548-688
495504198/249-379
AzNifL/1-137
EcAer/1-120
298708645/525-659
449453764/405-535
449525938/338-468
659124156/374-491
659124158/354-471
457693/32-163
148907121/24-158
224110804/231-357
675353822/143-266
367045084/271-390
302887177/281-415
635148931/37-181
635148933/52-185
635148707/587-736

Supplementary Figure 4. Extended alignment of natural Cys-less LOV* domains.

(a) Sequence alignment of VVD, BAT-LOV*, *B. subtilis* YtvA, *A. vinelandii* NifL, *E. coli* Aer, and LOV-like proteins lacking the adduct-forming cysteine. Tyr residues (yellow), Trp (blue), Cys (orange), and Met (green) are highlighted; circles above the alignment indicate the secondary structure in VVD with β sheets in blue and α helices in orange; arrows denote LOV-conserved residues, with the adduct-forming Cys in VVD and *B. subtilis* YtvA marked by a red arrow.

(b) Dendrogram of the alignment shown in **(a).**

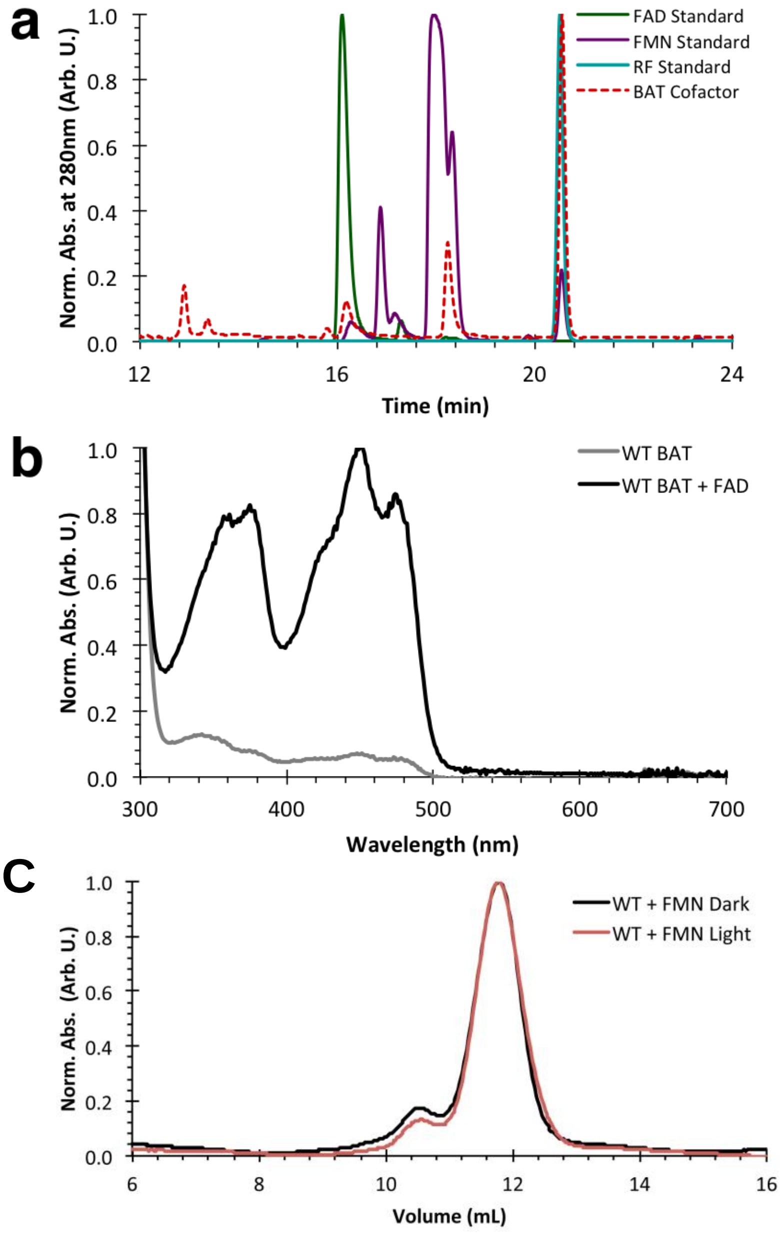

Supplementary Figure 5. Flavin binding and reconstitution of recombinantly expressed BAT-LOV*. (**a**) Reverse-phase HPLC trace of flavin moieties released from BAT-LOV* produced through recombinant expression in *E. coli.* BAT-LOV* primarily copurifies with riboflavin, although small amounts of FMN and FAD are also bound. (**b**) Absorbance spectra of WT BAT-LOV* before (grey) and after (black) reconstitution with FAD. Apoprotein separates from the flavin-bound protein on SEC (see Fig. 5d), and from these elution profiles, we estimate that 85-90% of BAT-LOV* can be reconstituted with either FAD or FMN. These estimates also match those based on flavin spectral quantification relative to protein absorption. (**c**) SEC traces of BAT-LOV* reconstituted with FMN in the dark and after broad-spectrum light exposure, as for the FAD-bound protein shown in Fig. 5d.

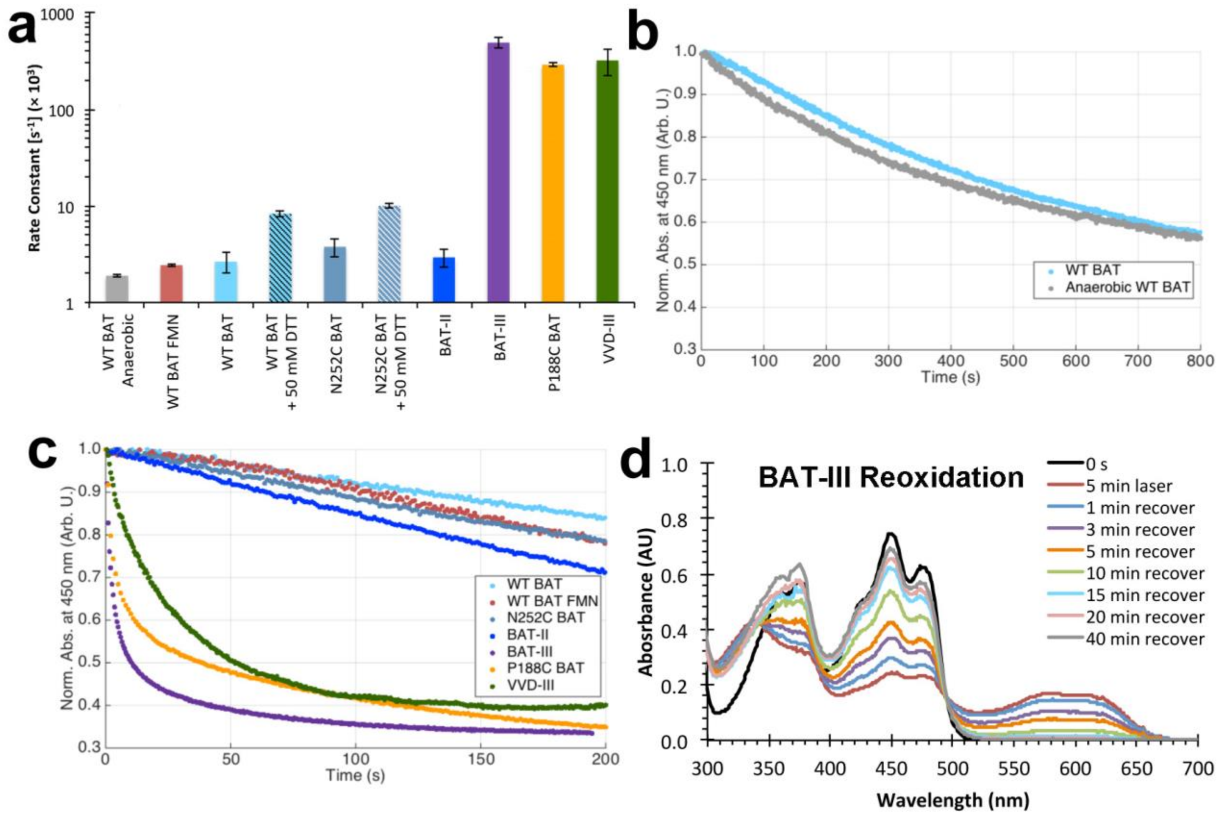

Supplementary Figure 6. Relative photoreduction yields of VVD-III and BAT-LOV*. (a) VVD-III reduces to the NSQ by orders of magnitude more rapidly than BAT-LOV* when exposed to 448-nm light. However, photoreduction yields of BAT-LOV* can be increased to rival those of VVD-III by either replacing the three flavin-proximal aromatic residues (BAT-III) with Phe or substituting the Pro residue that occupies the position which normally forms an adduct with a Cys residue (P188C). Addition of a Cys residue on Iβ ~13 Å from the flavin ring has only a small effect (N252C), as does addition of exogenous reductants. Reconstitution with FMN instead of FAD has an insignificant effect. (**b**) In the presence of air, there is a small lag phase for WT BAT-LOV* photoreduction that is not present under anaerobic conditions. (**c**) Time courses for photoreduction of VVD and BAT variants. (**d**) Recovery of photoreduced BAT-III to the oxidized state in aerobic solution.

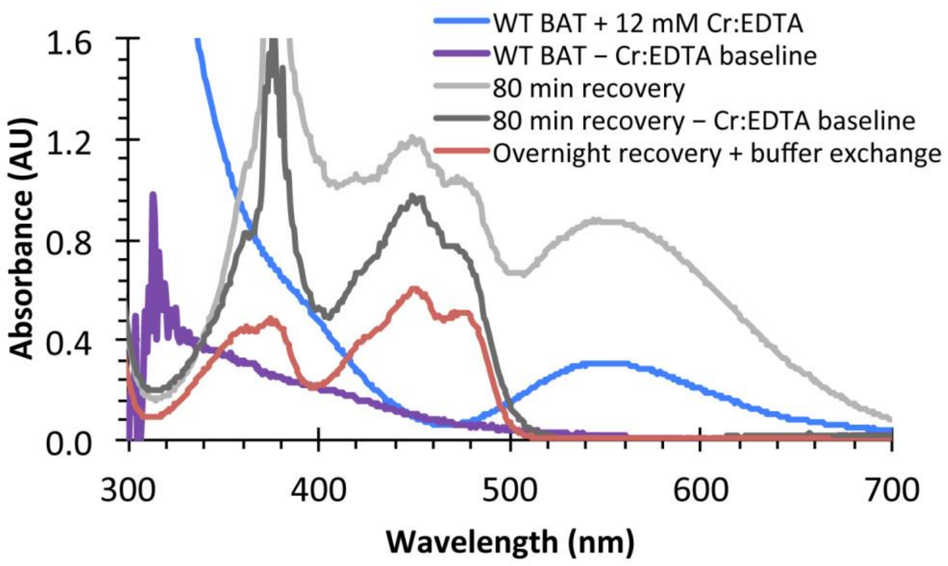

Supplementary Figure 7. Chemical reduction and recovery of BAT-LOV*. Treatment of BAT-LOV* with the low potential reductant Cr:EDTA generates a fully reduced flavin (blue line, broad absorption at 550 nm represents oxidation products of the Cr:EDTA reagent; purple line, with Cr:EDTA subtraction). Reoxidation after 80 min (shown with (dark grey) and without (light grey) Cr:EDTA subtraction) recovers the oxidized flavin, which is more evident after removal of the reductant by buffer exchange (red). A substantial amount of flavin remains bound to the protein after reoxidation.

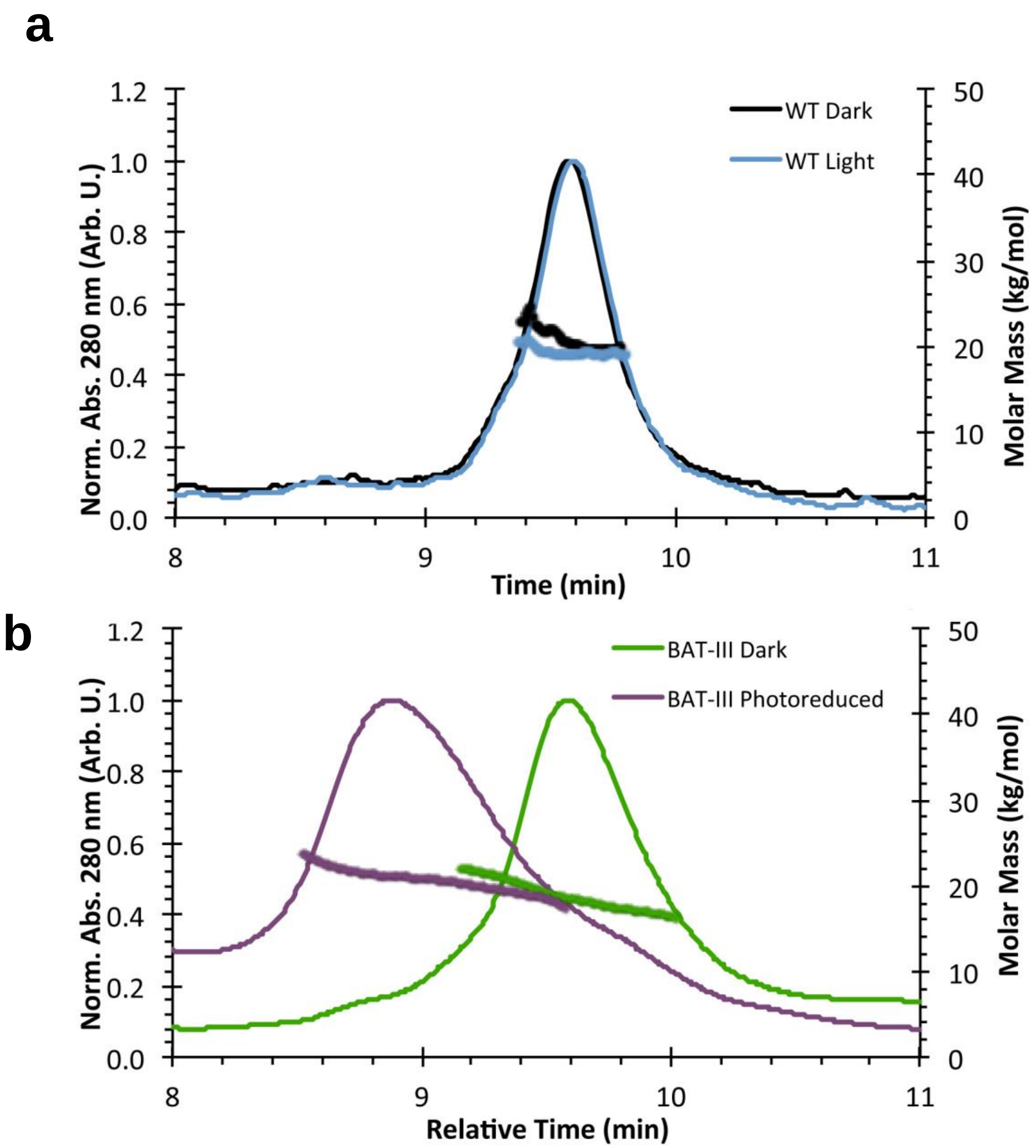

Supplementary Figure 8. Multi-angle light scattering of BAT-LOV* and BAT-III. (**a**) Molar mass measurements from MALS are shown across the SEC elution profiles for dark-adapted BAT-LOV*-FAD (black) and BAT-LOV* exposed to a 100 W broad-spectrum visible light source for 1.5 h prior to chromatography (blue). (**b)** MALS-SEC data of BAT-III in the dark-adapted and photoreduced state. The shift in BAT elution profile does not accompany a substantial change in molecular weight. Elution times are standardized to WT BAT-LOV*.

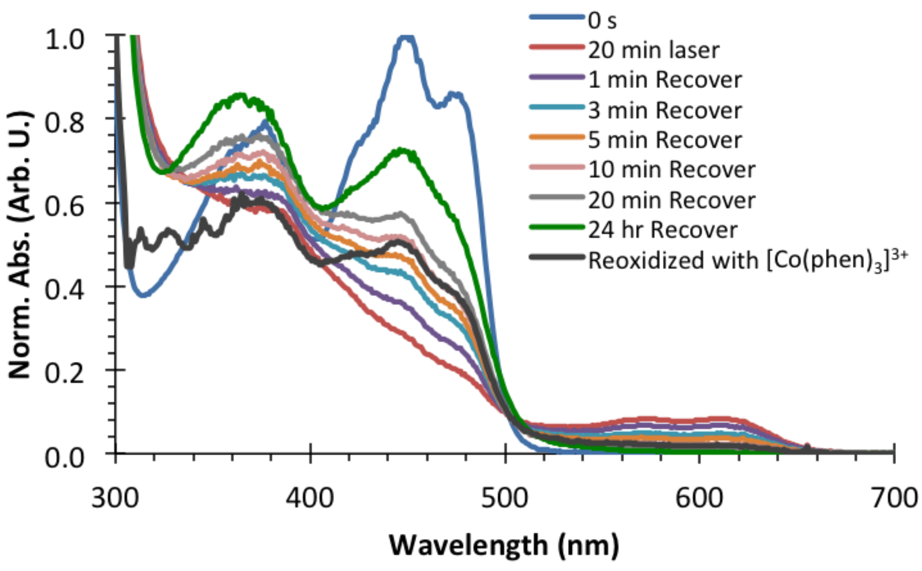

Supplementary Figure 9. Reoxidation of the BAT-P188C photoproduct. The spectrum of the light-excited protein strongly resembles that of a reduced HQ state. Furthermore, the light state can be rapidly converted to the oxidized flavin with the oxidant Co(III)$phen_3$. Over time, most of the reoxidized flavin dissociates from the protein (green trace). Some flavin is also unrecovered and presumably irreversibly photobleached. Unlike C4a adducts, the reduced state is also insensitive to bases, such as imidazole. In a 0.2-cm path length cuvette, 10 µL of 1 mM protein was photoreduced for 20 minutes under 448-nm laser light, after which, 1 µL of 1.2 mM $[Co(phen)_3](ClO_4)_3$ solution was immediately added. Spectra for the reoxidized species were collected within one minute (dark grey trace).
