# Supplementary material for: Signal transduction in light–oxygen–voltage receptors lacking the adduct-forming cysteine residue
Source: Nat Commun. 2015 Dec 9;6:10079. doi: 10.1038/ncomms10079 (PMC4682037; doi:10.1038/ncomms10079)
Supplement: Supplementary Data 1 — Overview of LOV-Like Proteins Lacking the Adduct-Forming Cysteine. The accompanying HTML File can be opened in a standard web browser and provides a graphical overview of the domain architecture of entries in GenBank that comprise LOV-Like domains lacking the adduct-forming cysteine residue. [file ncomms10079-s2.zip › 69190_2_data_set_1466514_nwvjvx.html]

Domain Architectures of Cysless LOV\* Proteins


## Domain Architectures of Cysless LOV\* Proteins

Supplementary material for Yee *et al.* "Signal Transduction in Light-Oxygen-Voltage Receptors Lacking the Adduct-Forming Cysteine Residue".
  
Note that the *Halorubrum hochstenium* bacterio-opsin activator (BAT) protein is entry "495857157".

---

Expand all
 
Collapse all

---

PAS\_9 has 18 occurrences.

"148907121" "302887177" "367045084" "490650830" "491104206" "491677185" "493940260" "494349052" "494809730" "495587172" "499541827" "504822023" "505221353" "506242629" "517068699" "541203563" "544601395" "558583472"

Response\_reg-PAS\_9-GAF\_2-HTH\_10 has 14 occurrences.

"155212693" "490734145" "491747656" "493056516" "493724709" "495269724" "495273460" "495279889" "495641236" "495715879" "495857157" "496124663" "515911956" "544602271"

PAS\_9-GGDEF-EAL has 6 occurrences.

"491548943" "496515604" "511072227" "552171632" "655089558" "675493957"

HAMP-PAS\_3-PAS\_9-PAS\_9-GGDEF-EAL has 4 occurrences.

"496533678" "496575888" "640546154" "653770099"

HAMP-PAS\_3-PAS\_3-PAS\_9-GGDEF-EAL has 4 occurrences.

"490765731" "501398515" "639204164" "640542378"

PAS\_9-HATPase\_c has 4 occurrences.

"144898713" "495889881" "499703955" "568205117"

PAS\_9-PAS\_9-Pkinase has 4 occurrences.

"449453764" "449525938" "659124156" "659124158"

PAS\_9-SpoIIE has 3 occurrences.

"491515482" "522052109" "670481149"

PAS\_9-GAF\_2-HTH\_10 has 3 occurrences.

"148753" "499205526" "558583704"

PAS\_9-GAF\_2-GGDEF-EAL has 3 occurrences.

"493883015" "495708415" "495772318"

Response\_reg-PAS-PAS\_4-PAS\_9-HisKA-HATPase\_c has 3 occurrences.

"491108658" "491119822" "495369233"

GAF\_2-PAS\_9-PAS\_9-GGDEF-EAL has 3 occurrences.

"491039211" "491156982" "588187355"

Hpt-Response\_reg-Response\_reg-PAS\_9-PAS\_9-PAS\_3-PAS-HisKA-HATPase\_c-Response\_reg has 3 occurrences.

"493948368" "652892586" "670487902"

Response\_reg-PAS\_9-PAS\_9-PAS\_4-HATPase\_c has 2 occurrences.

"494345671" "494348624"

PAS\_9-PAS\_9-HisKA-HATPase\_c has 2 occurrences.

"493681505" "504991123"

PAS\_4-GAF-PAS\_4-PAS\_3-PAS\_9-GAF-PAS\_3-HisKA\_2-HATPase\_c has 2 occurrences.

"652397765" "658300830"

PAS\_9-PAS\_9-GGDEF-EAL has 2 occurrences.

"515954762" "588678949"

PAS\_9-PAS\_9 has 2 occurrences.

"224110804" "675353822"

PAS\_4-PAS\_3-PAS\_9-GGDEF-EAL has 2 occurrences.

"499563902" "499697421"

PAS\_9-PAS\_9-PAS\_4-GGDEF-EAL has 2 occurrences.

"489552830" "648454791"

PAS\_9-PAS\_9-Pkinase-Pkinase has 2 occurrences.

"635148931" "635148933"

Response\_reg-PAS\_9-PAS\_4-PAS\_9-HisKA-HATPase\_c has 1 occurrence.

"494486825"

PAS\_4-GAF\_2-PAS\_9-GAF\_2-PAS\_9-GGDEF-EAL has 1 occurrence.

"251772029"

PAS\_9-PAS\_4-PAS\_9-SpoIIE has 1 occurrence.

"374852440"

PAS\_9-Pkinase has 1 occurrence.

"457693"

GAF\_3-PAS\_9-PAS-PAS\_9-PAS\_9-PAS\_3-PAS\_4-PAS\_3-PAS-PAS\_9-GAF\_3-PAS\_9-PAS\_9-PAS\_4-HATPase\_c has 1 occurrence.

"499544181"

GAF\_3-PAS\_9-PAS\_4-PAS\_9-PAS\_9-PAS\_3-PAS\_4-PAS\_3-HATPase\_c has 1 occurrence.

"505222758"

PAS\_3-PAS\_9-GGDEF has 1 occurrence.

"651235784"

Response\_reg-PAS\_9-HTH\_10 has 1 occurrence.

"493679590"

GAF-GAF-PAS\_9-MCPsignal has 1 occurrence.

"504955604"

PAS\_9-PAS\_4-PAS\_9-GAF\_3-PAS\_4-PAS\_3-HisKA-HATPase\_c has 1 occurrence.

"548767960"

CBS-PAS\_3-PAS\_3-PAS\_3-PAS\_9-GAF-PAS\_3-PAS\_3-PAS\_4-HisKA\_2-HATPase\_c has 1 occurrence.

"497470070"

PAS\_4-PAS\_3-PAS\_4-PAS\_3-PAS\_3-PAS\_9-HisKA\_2-HATPase\_c has 1 occurrence.

"516330201"

GAF\_3-PAS\_9-PAS\_9-HATPase\_c has 1 occurrence.

"492946794"

PAS\_9-PAS\_8-PAS\_4-PAS\_3-PAS\_9-GGDEF-EAL has 1 occurrence.

"516324372"

PAS\_9-PAS\_9-PAS\_9-PAS\_3-HisKA has 1 occurrence.

"505013918"

PAS\_9-PAS\_3 has 1 occurrence.

"682411003"

CBS-CBS-GAF-PAS\_3-PAS\_3-PAS\_3-PAS\_9-GAF-PAS\_3-PAS\_3-HisKA\_2-HATPase\_c has 1 occurrence.

"553730274"

RGS-PAS\_9-PAS\_9-PAS\_9 has 1 occurrence.

"298708645"

CBS-PAS\_9-PAS\_3-PAS\_4-PAS\_9-PAS\_3-HisKA-HATPase\_c has 1 occurrence.

"497074813"

PAS\_2-GAF-PHY-PAS\_9-PAS\_9-Pkinase has 1 occurrence.

"635148707"

PAS\_4-PAS\_9-GGDEF-EAL has 1 occurrence.

"517214330"

PAS\_9-GGDEF has 1 occurrence.

"503059604"

PAS\_9-PAS\_9-PAS\_9-HATPase\_c has 1 occurrence.

"496124150"

CBS-PAS\_9-PAS\_3-PAS\_4-PAS\_9-HisKA-HATPase\_c has 1 occurrence.

"515878998"

PAS\_9-PAS\_9-PAS\_9-HATPase\_c-Response\_reg has 1 occurrence.

"495504198"

Response\_reg-PAS\_8-PAS\_9-GGDEF-EAL-Response\_reg has 1 occurrence.

"517848989"

---

generated with autoblast by A.M. 2015
